# Fucoxanthin diminishes oxidative stress damage in human placenta-derived mesenchymal stem cells through the PI3K/Akt/Nrf-2 pathway

**DOI:** 10.1038/s41598-023-49751-5

**Published:** 2023-12-27

**Authors:** Gunticha Suwanmanee, Chairat Tantrawatpan, Pakpoom Kheolamai, Luminita Paraoan, Sirikul Manochantr

**Affiliations:** 1 Division of Cell Biology, Department of Preclinical Sciences, Faculty of Medicine, Thammasat University, Pathumthani, 12120 Thailand; 2 Center of Excellence in Stem Research and Innovation, Thammasat University, Pathumthani, 12120 Thailand; 3 Department of Biology, Faculty of Arts and Sciences, Edge Hill University, BioSciences Building, St Helens Road, Ormskirk, L39 4QP UK

**Keywords:** Biochemistry, Cell biology, Molecular biology, Stem cells, Structural biology, Molecular medicine

## Abstract

Placenta-derived mesenchymal stem cells (PL-MSCs) have therapeutic potential in various clinical contexts due to their regenerative and immunomodulatory properties. However, with increasing age or extensive in vitro culture, their viability and function are gradually lost, thus restricting their therapeutic application. The primary cause of this deterioration is oxidative injury from free radicals. Therefore, enhancing cell viability and restoring cellular repair mechanisms of PL-MSCs in an oxidative stress environment are crucial in this context. Fucoxanthin, a carotenoid derived from brown seaweed, demonstrates antioxidant activity by increasing the production of antioxidant enzymes and lowering the levels of reactive oxygen species (ROS). This study aimed to determine whether fucoxanthin protects PL-MSCs from hydrogen peroxide (H_2_O_2_)-induced oxidative stress. After characterization, PL-MSCs were co-treated with fucoxanthin and H_2_O_2_ for 24 h (co-treatment) or pre-treated with fucoxanthin for 24 h followed by H_2_O_2_ for 24 h (pre-treatment). The effects of fucoxanthin on cell viability and proliferation were examined using an MTT assay. The expression of antioxidant enzymes, PI3K/Akt/Nrf-2 and intracellular ROS production were investigated in fucoxanthin-treated PL-MSCs compared to the untreated group. The gene expression and involvement of specific pathways in the cytoprotective effect of fucoxanthin were investigated by high-throughput NanoString nCounter analysis. The results demonstrated that co-treatment and pre-treatment with fucoxanthin restored the viability and proliferative capacity of PL-MSCs. Fucoxanthin treatment increased the expression of antioxidant enzymes in PL-MSCs cultured under oxidative stress conditions and decreased intracellular ROS accumulation. Markedly, fucoxanthin treatment could restore PI3K/Akt/Nrf-2 expression in H_2_O_2_-treated PL-MSCs. High-throughput analysis revealed up-regulation of genes involved in cell survival pathways, including cell cycle and proliferation, DNA damage repair pathways, and down-regulation of genes in apoptosis and autophagy pathways. This study demonstrated that fucoxanthin protects and rescues PL-MSCs from oxidative stress damage through the PI3K/Akt/Nrf-2 pathway. Our data provide the supporting evidence for the use of fucoxanthin as an antioxidant cytoprotective agent to improve the viability and proliferation capacity of PL-MSCs both in vitro and in vivo required to increase the effectiveness of MSC expansion for therapeutic applications.

## Introduction

Mesenchymal stem cells (MSCs) are multipotent cells capable of differentiating into adipocytes, chondrocytes, and osteoblasts^1^. In addition to supporting hematopoietic stem cells in the bone marrow, MSCs possess immunomodulatory and regenerative properties in injured tissues, suppress inflammatory damage, and contribute to tissue repair^2^. MSCs are rare in vivo and their number in bone marrow decreases with age^3^. Furthermore, the proliferative and differentiated potential of MSCs from aging donors also decreases. MSCs that reside in connective tissue, such as adipose tissue, may be affected by aging^4^ and primarily affect the restoration process in the aging population^5^.

MSCs required extensive ex vivo expansion to reach a sufficient number for therapeutic application. However, the replicative senescence of MSCs limits the capacity for cell division^6^. Replicative senescence also negatively affects their immunomodulatory and differentiation functions, as well as their potential activity against graft-versus-host disease (GvHD) and other inflammatory pathologies^7^. Age-related alterations in MSCs are influenced by both genetic and local microenvironmental factors^6^. The results of telomere shortening, accumulation of oxidative damage, impaired DNA repair, altered gene expression profiles, and epigenetic alterations of aging can cause cellular dysfunctions^8^. However, chronological aging of MSCs in vivo has been shown to have overlapping and non-overlapping features with in vitro aging^9^. Poor cell viability in injured tissue caused by a hypoxic microenvironment and related to oxidative stress appears to be the primary limitation of MSC-based therapies^10^.

Oxidative stress is caused by the increase of reactive oxygen species (ROS) exceeding the physiological thresholds, damaging cell membranes, proteins, and DNA. It is recognized as a primary factor for decreasing in vivo and in vitro MSC survival, resulting in a low survival rate of transplanted MSCs^11,12^. Therefore, approaches to reduce common denominators that cause cellular damage are required to increase the viability of MSCs and restore their cellular repair mechanisms.

Fucoxanthin, a marine carotenoid extracted from brown seaweeds, is considered a potent antioxidant due to its unique biologically active structure. It is an essential bioactive substance with several bioactivities including anti-obesity, anti-cancer and anti-inflammation^13,14^. Fucoxanthin decreases ROS levels, inhibits DNA damage, restores mitochondrial membrane potential, and suppresses apoptosis in human keratinocytes^15,16^. *Undaria pinnatifida* contains several beneficial substances, including fucoxanthin^17^. Bone marrow-derived MSCs (BM-MSCs) treated with *U. pinnatifida* extract exhibited elevated expression of the antioxidant enzymes superoxide dismutase (SOD) and catalase (CAT)^18^. Fucoxanthin has cytoprotective effects against hydrogen peroxide-induced oxidative damage in human liver L02 cells by decreasing the intracellular ROS content and increasing the intracellular reduced glutathione (GSH)^19^.

The phosphoinositide 3-kinase (PI3K)/protein kinase B (Akt) signaling pathway plays central regulatory roles in the survival, proliferation, migration, and differentiation of MSCs^20,21^. PI3K/Akt signaling is involved in the survival pathway by modulating the expression of numerous antioxidant proteins, including nuclear factor erythroid 2-related factor 2 (Nrf-2), SOD, GSH and CAT^22–24^.

MSCs can be obtained from various sources including adipose tissue, bone marrow, umbilical cord, placenta, and amniotic membrane^25–29^. As a discarded biological material, the placenta is a particularly rich source of MSCs. MSCs can be isolated from the placenta using a non-invasive technique and can be prepared in large quantities for clinical applications. Importantly, placenta-derived MSCs (PL-MSCs) acquire the characteristics of MSCs as defined by the International Society for Cell & Gene Therapy (ISCT) similar to MSCs derived from the bone marrow (BM-MSCs). A previous study reported that the clonogenicity and function of PL-MSCs were superior to those derived from BM-MSCs^30,31^ thus supporting their potential for use in regenerative applications. However, the effectiveness of their in vitro expansion, measured by survival and proliferation, requires further development. Enhancing the in vivo longevity of MSCs is also crucial.

Fucoxanthin may act as a powerful modulator of MSCs through its antioxidant activities. Such applications would require characterization of the effect of fucoxanthin on PL-MSCs under oxidative stress conditions, which is poorly understood. Since there is currently limited data on the cytoprotective effects of fucoxanthin on PL-MSCs, the purpose of this study was to examine the effect and underlying mechanism of fucoxanthin on the survival and proliferation of PL-MSCs under oxidative stress conditions. The data obtained provides an additional strategy for preventing oxidative stress-induced damage to MSCs in both in vivo and in vitro models.

## Materials and methods

**Figure 1:**
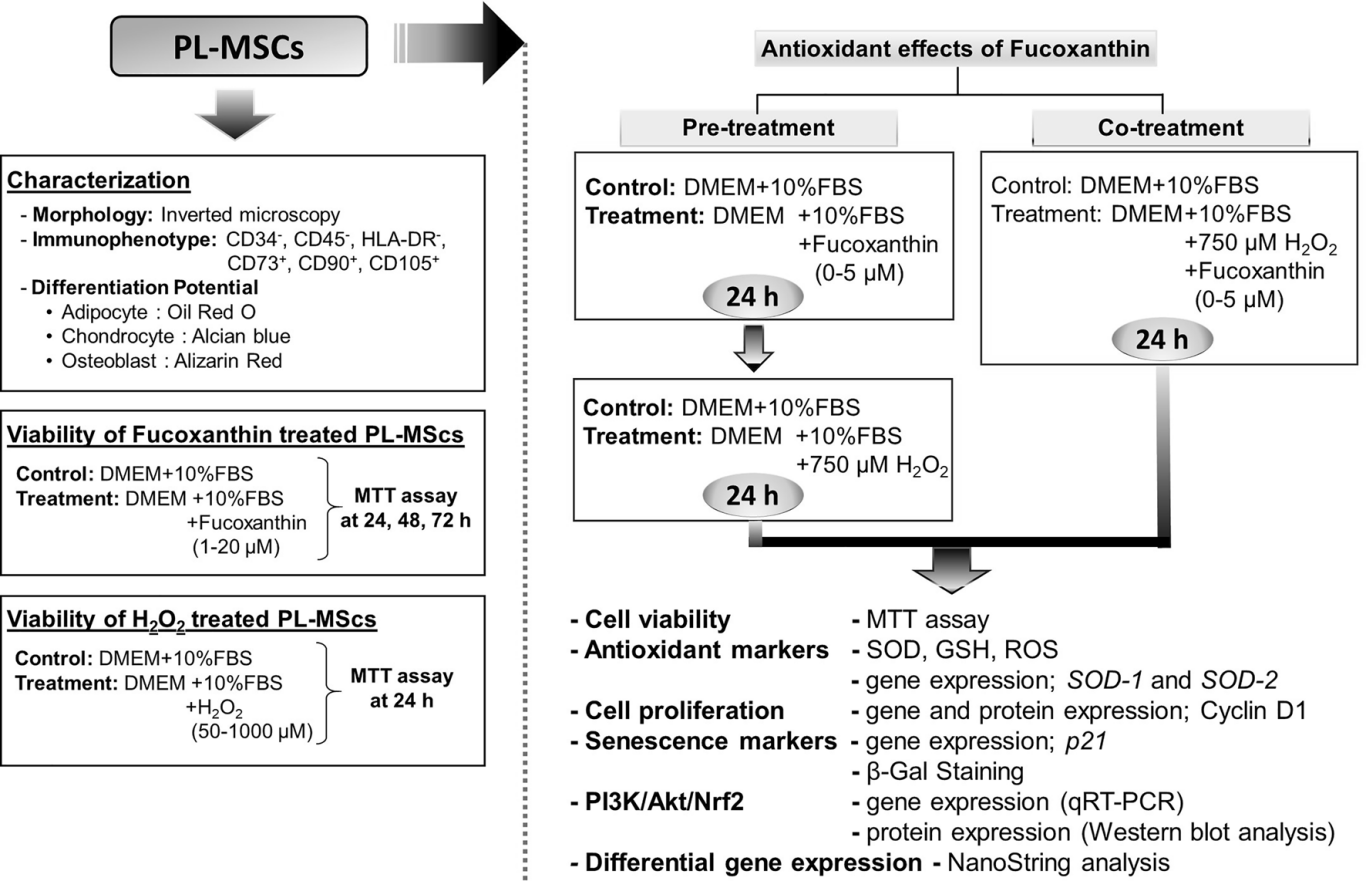
A schematic diagram illustrates the overall experimental schedule.

An overall experimental schedule was illustrated in the following diagram (Fig. 1).

### Cell isolation and culture

The study was approved by the Human Ethics Committee of Thammasat University (Medicine) [Approval number: 024/2021]. After normal deliveries at the Thammasat Chalermprakiat Hospital, placental tissues of five healthy volunteers were collected. The tissues were cut into small pieces and digested at 37 °C for 2 h with 0.5% (w/v) trypsin–EDTA (GibcoBRL, USA). The digested tissues were then cultured with completed DMEM medium [Dulbecco's modified Eagle's medium (Gibco-BRL, USA) supplemented with 10% fetal bovine serum (FBS, GibcoBRL, USA), 2 mM Glutamax™, 100 U/ml of penicillin and 100 µg/ml streptomycin (both from GibcoBRL)]. The cell cultures were maintained at 37 °C in a humidified atmosphere with 5% carbon dioxide (CO_2_). The medium was changed every 3–4 days. At 70–80% confluence, the MSCs were trypsinized and passaged using 0.25% w/v trypsin/EDTA (GibcoBRL, USA).

### Characterization of MSCs

The cells from placenta were classified as MSCs using the following criteria: their ability to adhere to the plastic surface, positive and negative cell surface markers, and their propensity to differentiate into many cell types.

#### Immunophenotypical assay

Trypsinized PL-MSCs were suspended in phosphate buffered saline (PBS). Then, 5 × 10^5^ MSCs in 50 µl of PBS were incubated with fluorescein isothiocyanate (FITC) or phycoerythrin (PE) conjugated antibodies against various human antigens, including HLA-DR-PE (Bio Legend, USA) cluster of differentiation (CD) 34-PE (Bio Legend, USA), CD45-FITC (Bio Legend, USA), CD73-PE (Bio Legend, USA), CD90-PE (BD Biosciences, USA) and CD105-PE (BD Biosciences, USA) for 30 min at 4 °C in the dark. Cells were fixed with 1% paraformaldehyde in PBS after rinsing with PBS. At least 2 × 10^4^ labeled cells were acquired and analyzed from each sample using flow cytometry (DxFLEX flow cytometer, Beckman Coulter, USA) and CytExpert software (DxFLEX flow cytometer, Beckman Coulter, USA).

#### The differentiation assay

For osteogenic differentiation, PL-MSCs from passages 3 to 5 were seeded at a density of 4.5 × 10^3^ cells/cm^2^ and allowed to adhere to 6-well cell culture plates (Corning, USA) overnight. Subsequently, the medium was discarded and replaced with osteogenic differentiation medium [completed DMEM medium supplemented with 100 nM dexamethasone and 50 µM ascorbic acid 2-phosphate (Sigma-Aldrich, USA)]. The addition of 10 mM β-glycerophosphate (Sigma-Aldrich, USA) was performed on day 7 of induction. On day 28 of the culture, cells were stained for 30 min with 40 mM alizarin red S (Sigma-Aldrich, USA). The calcifications in differentiated cells were observed under an inverted microscope (Nikon Eclipse Ts2R, Japan).

For adipogenic differentiation, PL-MSCs were trypsinized and cultured with complete DMEM medium at a density of 4.5 × 10^3^ cells/cm^2^ in 6-well plates overnight. The medium was then changed to the adipogenic differentiation medium [completed DMEM medium supplemented with 100 µM indomethacin, 25 mM glucose, 1 µM dexamethasone, and 1 µg/ml insulin (all from Sigma-Aldrich, USA)] for 28 days. Cells were stained for 20 min with 0.5% Oil Red O (Sigma-Aldrich, USA) in 6% isopropanol. The lipid droplets in differentiated cells were visualized under an inverted microscope (Nikon Eclipse Ts2R, Japan).

For chondrogenic differentiation, PL-MSCs were seeded at a density of 3 × 10^6^ cells/cm^2^ in 96-well U-bottom cell culture plates (Jet Biofil, China). Cells were incubated in complete DMEM medium at 37 °C in a humidified atmosphere with 5% CO_2_ overnight. The medium was then substituted with a complete MSCgo™ Chondrogenic XF medium (Sartorius, Germany). After 3 weeks of induction, the spheroidal mass was fixed with 10% formalin for 30 min at room temperature before being stained overnight in the dark with 1% Alcian Blue Solution (HiMedia, India) at room temperature. The stained mass was observed under an inverted microscope (Nikon Eclipse Ts2R, Japan). For controls, MSCs were cultured in complete DMEM medium and processed similarly to cells in each differentiation medium, respectively.

### Cell viability assay

The viability of fucoxanthin-treated PL-MSCs was evaluated by the 3-(4, 5-dimethylthiazol-2-yl)-2,5-diphenyltetrazolium bromide (MTT) assay (Sigma-Aldrich, USA). Briefly, PL-MSCs were seeded at a density of 3 × 10^4^ cells/cm^2^ into a 96-well plate (Corning, USA). After overnight culture, cells were treated with fucoxanthin (Sigma-Aldrich, USA) at final concentrations of 1, 2, 3, 4, 5, 10, and 20 µM for 24, 48 and 72 h. Following the respective experimental time, the cells were incubated with 0.5 mg/ml MTT in completed DMEM for 4 h at 37 °C. Subsequently, 100 μl dimethyl sulfoxide (DMSO) was added to each well. The absorbance was measured at 570 nm using a Synergy HT Multi-Detection Microplate Reader and Agilent BioTek Gen6 software (BioTek Instruments Inc., USA). Cell viability was calculated as shown in the equation:

$$ {\text{Cell }}\;{\text{viability }}\left( {\% {\text{ of }}\;{\text{control}}} \right) = \left( {\frac{{ {\text{OD }}\left( {{\text{test}}} \right) \, - {\text{ OD }}\left( {{\text{blank}}} \right) }}{{{\text{OD }}\left( {{\text{control}}} \right) \, - {\text{ OD }}\left( {{\text{blank}}} \right)}}} \right) \times 100 $$

### H_2_O_2_-induced cytotoxicity assay

PL-MSCs were seeded into a 96-well plate (Corning, USA) at a density of 3 × 10^4^ cells/cm^2^ and incubated at 37 °C in the presence of 5% CO_2_ for 24 h. After removing the medium, the PL-MSCs were incubated with various concentrations of H_2_O_2_ (50–1000 µM) in complete DMEM medium at 37 °C and 5% CO_2_ for 24 h. Control cells were cultured in complete DMEM medium without H_2_O_2_. Cell viability was examined using an MTT assay. The absorbance was measured at 570 nm using a Synergy HT multi-detecting microplate reader as described above.

### The effect of fucoxanthin on the viability of H_2_O_2_-treated PL-MSCs

To evaluate the protective effect of fucoxanthin on MSC viability under oxidative stress conditions, PL-MSCs were seeded in a 96-well plate (Corning, USA) at a density of 3 × 10^4^ cells/cm^2^. Two treatment groups were designed: (1) Co-treatment: PL-MSCs were treated with the combination of 750 µM H_2_O_2_ and 1–5 µM fucoxanthin for 24 h, and (2) Pre-treatment: PL-MSCs were pre-treated with fucoxanthin at a concentration of 1–5 µM for 24 h; subsequently, the medium was removed and PL-MSCs were incubated with fresh complete DMEM medium supplemented with 750 µM H_2_O_2_ without fucoxanthin for 24 h. PL-MSCs cultured in a complete DMEM medium served as a control. The viability of PL-MSCs was evaluated using an MTT assay, and the absorbance was measured at 570 nm using a Synergy HT multi-detecting microplate reader as described above.

### Cellular senescence assay

The investigation of replicative senescence of PL-MSCs under oxidative stress conditions was conducted using a β-galactosidase (β-Gal) activity assay kit (Sigma-Aldrich, USA) following the instructions provided by the manufacturer. The PL-MSCs were seeded into a 12-well plate (Corning, USA) at a density of 1 × 10^4^ cells/cm^2^ and kept in an incubator at a temperature of 37 °C with a 5% CO_2_ for 24 h. Subsequently, PL-MSCs underwent co-treatment or pre-treatment with fucoxanthin at concentrations ranging from 1 to 5 µM and H_2_O_2_ at a concentration of 750 µM for 24 h, as previously mentioned. PL-MSCs cultured in a complete DMEM medium served as a control. The PL-MSCs were subsequently fixed using a 1X fixative buffer for 7 min at ambient temperature. Afterwards, the PL-MSCs were cultured with a substrate, 5-bromo-4-chloro-3-indolyl-β-d-galactopyranoside (X-gal), pH 6.0, for 6 h at 37 °C. The identification of the blue color resulting from β-Gal staining was performed using an inverted microscope (Nikon Eclipse Ts2R, Japan). The cells exhibiting a positive blue color were enumerated, along with the total number of cells in each sample, across 9 fields. The data were calculated and expressed as a percentage of senescent cells using the following formula:

$$ {\text{Senescent}}\;{\text{ cells }}\left( \% \right) = \frac{{\left( {{\text{Number}}\;{\text{of}}\;{\text{positive}}\;{\text{cells}}} \right)}}{{\left( {{\text{Number}}\;{\text{of}}\;{\text{total}}\;{\text{cells}}} \right)}} \times 100 $$

### Detection of antioxidant biomarkers

The antioxidant biomarkers SOD and GSH were investigated using a colorimetric SOD activity assay kit (Sigma-Aldrich, USA) and a GSH + GSSG/GSH assay kit (Abcam, USA) according to the manufacturer's instructions, respectively.

#### Superoxide dismutase (SOD) activity assay

PL-MSCs were seeded at a density of 1.5 × 10^4^ cells/cm^2^ into a 6-well plate (Corning, USA) and incubated at 37 °C under 5% CO_2_ for 24 h. Then, PL-MSCs were co-treated or pre-treated with 1–5 µM fucoxanthin and 750 µM H_2_O_2_ for 24 h as described above. PL-MSCs cultured in complete DMEM medium served as control. Subsequently, the PL-MSCs were harvested and lysed with lysis buffer (0.1 M glycine, 1% Nonidet P-40, 1 mM MgCl_2_ and 1 mM ZnCl_2_, pH 9.6). After centrifugation at 8000×g, 4 °C for 10 min, the supernatants were collected for the colorimetric SOD activity assay according to the manufacturer's instructions. The absorbance was measured at 440 nm using a Synergy HT Multi-Detection Microplate Reader. Units of SOD activity in the sample solution were determined using the standard SOD enzyme (Sigma-Aldrich, USA). The measured SOD activity was expressed as units/mg of protein.

#### Glutathione (GSH) assay

PL-MSCs were seeded at a density of 1.5 × 10^4^ cells/cm^2^ into each well of 6-well plates (Corning, USA) and incubated for 24 h at 37 °C under 5% CO_2_. The PL-MSCs were then co-treated or pre-treated with 1–5 µM fucoxanthin and 750 µM H_2_O_2_ for 24 h. The control was cultured in complete DMEM medium only. Subsequently, the PL-MSCs were collected and lysed with 80 µl glutathione buffer. The cells were then incubated on ice for 10 min and 20 µl of 5% sulfosalicylic acid (SSA) was added. After centrifuging at 8000 × g, 4 °C for 10 min, the supernatant was collected. The yellow-colored substrate was measured at 405 nm using a Synergy HT multi-detecting microplate reader. The concentration of GSH in the sample solution was determined using the standard glutathione calibration curve.

### Detection of ROS as an oxidative stress biomarker

The expression of intracellular ROS was evaluated by measuring the oxidative conversion of cell-permeable 2′, 7′-Dichlorofluorescein diacetate (DCFH-DA; Abcam, USA) to fluorescent dichlorofluorescein (DCF) following the manufaturer's instructions using a fluorospectrophotometer and fluorescence microscopy. For fluorospectrophotometer, PL-MSCs were seeded at a density of 3 × 10^4^ cells/cm^2^ into each well of a 96-well black clear bottom plate (SPL Life Science, South Korea). For fluorescence visualization, PL-MSCs were seeded at a density of 3 × 10^4^ cells/cm^2^ into each well of a 24-well plate (Corning, USA). The PL-MSCs were co-treated or pre-treated with fucoxanthin at a concentration of 1–5 µM and 750 µM H_2_O_2_ as described above. PL-MSCs cultured in complete DMEM medium served as control. At the respective experimental time, the cells were washed with PBS and incubated with 20 µM DCFH-DA in PBS at 37 °C for 1 h. Intracellular ROS converted non-fluorescent DCFH-DA molecules into fluorescent DCF molecules. Subsequently, the fluorescent intensities were determined using a fluorescent microplate reader (Varioskan™ LUX multimode microplate reader; Thermo Fisher Scientific, USA) at an excitation wavelength of 495 nm and an emission wavelength of 529 nm. For visualization, representative images of intracellular ROS levels visualized through green fluorescent labeling and nuclear staining with Hoechst 33342 exhibiting blue fluorescence were also captured using inverted fluorescence microscopy (Nikon Eclipse Ts2R, Japan).

### Gene expression analysis by quantitative real-time polymerase chain reaction (qRT-PCR)

To investigate the expression of genes in PL-MSCs cultured under oxidative stress conditions, PL-MSCs were co-treated or pre-treated with 1–5 µM fucoxanthin and 750 µM H_2_O_2_ for 24 h and 48 h. PL-MSCs cultured in complete DMEM medium served as control. At each time point, total RNA was extracted using TRIzol® reagent (Invitrogen, USA). Then, 1 µg of extracted RNA was reverse transcribed into cDNA using iScript™ Reverse Transcription supermix (Bio-Rad, USA) according to the manufacturer’s instructions. Gene expression was quantified using iTaq™ Universal SYBR® green supermix (Bio-Rad, USA). Thermocycling conditions were as follows: pre-denature at 95 °C for 10 min, followed by 40 cycles of denaturation at 95 °C for 15 s and annealing/amplification at 60 °C for 60 s using the StepOne Plus® real-time PCR system (Applied Biosystems, USA). All reactions were performed at least in triplicate and analyzed using StepOne™ Software version 2.3 (Applied Biosystems, USA). Target gene expression levels were normalized against glyceraldehyde-3-phosphate dehydrogenase (*GAPDH*) based on the relative quantification formula of 2^−ΔΔCt^. The primer sequences are listed in Table 1.

**Table 1:** Sequence of the primers used for qRT‑PCR.

Gene	Forward primer	Reserve primer	Product size (bp)
*p21*	5′-TTTGGCTCCCCTGTACCTTT-3′	5′-CCTTCCCCTTCCAGTCCATT-3′	192
*SOD-1*	5′-GAAGGTGTGGGGAAGCATTA-3′	5′-ACATTGCCCAAGTCTCCAAC-3′	174
*SOD-2*	5′-GGAAGCCATCAAACGTGACT-3′	5′-CCTTGCAGTGGATCCTGATT-3′	162
*CCND1*	5′-CGTGGCCTCTAAGATGAAGG-3′	5′-CTGGCATTTTGGAGAGGAAG-3′	184
*PI3K*	5′-CAATCGGTGACTGTGTGGGA-3′	5′-ACAGGTCAATGGCTGATCA-3′	170
*Akt*	5′-CAAGTCCTTGCTTTCAGGGC-3′	5′-ATACCTGGTGTCAGTCTCCGA-3′	183
*Nrf-2*	5′-CGGTATGCAACAGGACATTG-3′	5′-ACTGGTTGGGGTCTTCTGTG-3′	103
*GAPDH*	5′-CAATGACCCCTTCATTGACC-3′	5′-TTGATTTTGGAGGGATCTCG-3′	159

### Protein extraction and Western blot analysis

Expressions of cyclin D1, an essential protein for cell cycle progression, and the PI3K/Akt/Nrf-2 signaling pathway in PL-MSCs cultured under oxidative stress conditions as above were investigated by Western blot analysis. Cells were lysed with RIPA buffer (0.05 M Tris–HCl, pH 7.4, 1% TritonX-100, 1% sodium deoxycholate, 0.1% SDS, 0.15 M NaCl) containing a protease and phosphatase inhibitor cocktail (Cell Signaling Technology, USA). After incubation for 20 min on ice, the lysates were centrifuged to remove cell debris. Protein concentration was determined using the Bradford assay (Bio‑Rad Laboratories, Inc.). For Western blot analysis, equal amounts of protein (20 µg) from each sample diluted with the 3X reducing SDS loading buffer (Cell Signaling Technology, USA), and a molecular weight marker (Abcam, USA) were loaded onto 12% sodium dodecyl sulfate polyacrylamide gel electrophoresis (SDS-PAGE). Electrophoresis was carried out at 120 V for 90 min. Proteins on SDS-PAGE gels were transferred to nitrocellulose membranes (0.45 µm pore-size; Bio-Rad, USA) using a Mini Trans-Blot® Electrophoretic Transfer Cell (Bio-Rad, USA) at 120 V for 90 min.

Following transfer, the membranes were gently stained using 0.1% (w/v) Ponceau S in 5% acetic acid. Next, the membranes were carefully cut to facilitate the incubation process with different primary antibodies. Afterward, the membranes were blocked with 5% non-fat dry milk in Tris buffered saline with 0.1% Tween® 20 (TBST) for 1 h. Subsequently, the membranes were incubated with primary antibodies (Table 2) at 4 °C overnight and incubated with secondary antibodies, horseradish peroxidase (HRP)-conjugated mouse anti-rabbit antibody (1:10,000 dilution; Jackson ImmunoResearch, USA) or goat anti-mouse antibody (1:10,000 dilution; Jackson ImmunoResearch, USA) for 1 h at room temperature. The protein bands were detected with enhanced chemiluminescence (ECL) using Clarity™ Western ECL Substrate (Bio-Rad, USA). The signals were captured with an Amersham Imager 600 (GE Healthcare Life Sciences). For quantification, the protein band intensity was quantified using NIH software (ImageJ) and expressed as a ratio to the β-actin band intensity.

**Table 2:** Characteristics of the primary antibodies used.

Antibody	Host	Dilution	Company
**Cell cycle**			
Cyclin D1	Mouse	1:5000	Proteintech, USA
**PI3K signaling pathway**			
PI3 Kinase p85	Rabbit	1:1000	Cell Signaling Technology, USA
Phospho-PI3 kinase p85	Rabbit	1:1000	Cell Signaling Technology, USA
Akt	Rabbit	1:1000	Cell Signaling Technology, USA
Phospho-Akt	Rabbit	1:1000	Cell Signaling Technology, USA
Nrf-2	Rabbit	1:1000	Cell Signaling Technology, USA
β-actin	Mouse	1:10,000	Proteintech, USA

### Nanostring® nCounter assay

To investigate the effect of fucoxanthin on gene expression in H_2_O_2_-treated PL-MSCs, NanoString® nCounter Technology (NanoString Technologies, Seattle, WA, USA) was performed using the nCounter® metabolic pathways panel. PL-MSCs were co-treated with a complete medium supplemented with 5 µM fucoxanthin and 750 µM H_2_O_2_ for 24 h. The controls were cultured in complete medium and complete medium supplemented with 750 µM H_2_O_2_ for 24 h. Total RNA was isolated using the PureLink™ RNA Mini Kit (Ambion, USA). One hundred nanograms of extracted mRNA were used as input material. Subsequently, a hybridization process was conducted overnight at a temperature of 65 °C, utilizing 50 bases of nCounter Reporter and Capture probes. Following the process of hybridization, the samples were subsequently introduced into the nCounter Prep Station for the purpose of purifying the samples and immobilizing the target/probe complex onto the cartridge. The nCounter Digital Analyzer was utilized to perform a high-density scan (555 fields of view) for each assay. This scan was designed to enumerate individual fluorescent barcodes and measure the abundance of target RNA molecules in each sample. Analysis of multiplexed gene expression of 768 genes was performed following the manufacturer’s instructions on the Counter Flex system and using the nSolver software v4.0. Transcription copies were standardized using the geometric mean of 20 maintenance genes for baseline and normalization. The threshold count value of 50 was the baseline subtraction parameter; gene expression fold changes were calculated by comparing fucoxanthin-treated samples with untreated controls. Raw P values from differential expression analyzes were used to evaluate gene expression data. All heat maps and data sets were generated using the nCounter Analysis and Advanced Analysis packages of nSolver4.0 (NanoString Technologies, Seattle, WA, USA).

### Statistical analysis

Data were analyzed and presented as the mean ± standard error of the mean (SEM). Statistical comparisons were performed using the paired T-test for paired samples, using SPSS software version 25 (SPSS, Inc.). A *p*-value of less than 0.05 was considered statistically significant.

### Ethics approval and consent to participate

All experimental procedures were conducted in accordance with the Declaration of Helsinki and the Belmont report. This study was approved by the Human Research Ethics Committee of Thammasat University (Medicine) [Approval title: The effects of fucoxanthin on the proliferation and osteogenic differentiation of human mesenchymal stem cells.] [Approval number: 024/2021] (Date of approval: February 4, 2021). All samples were obtained from donors with written informed consent.

## Results

### Characterization of PL-MSCs

**Figure 2:**
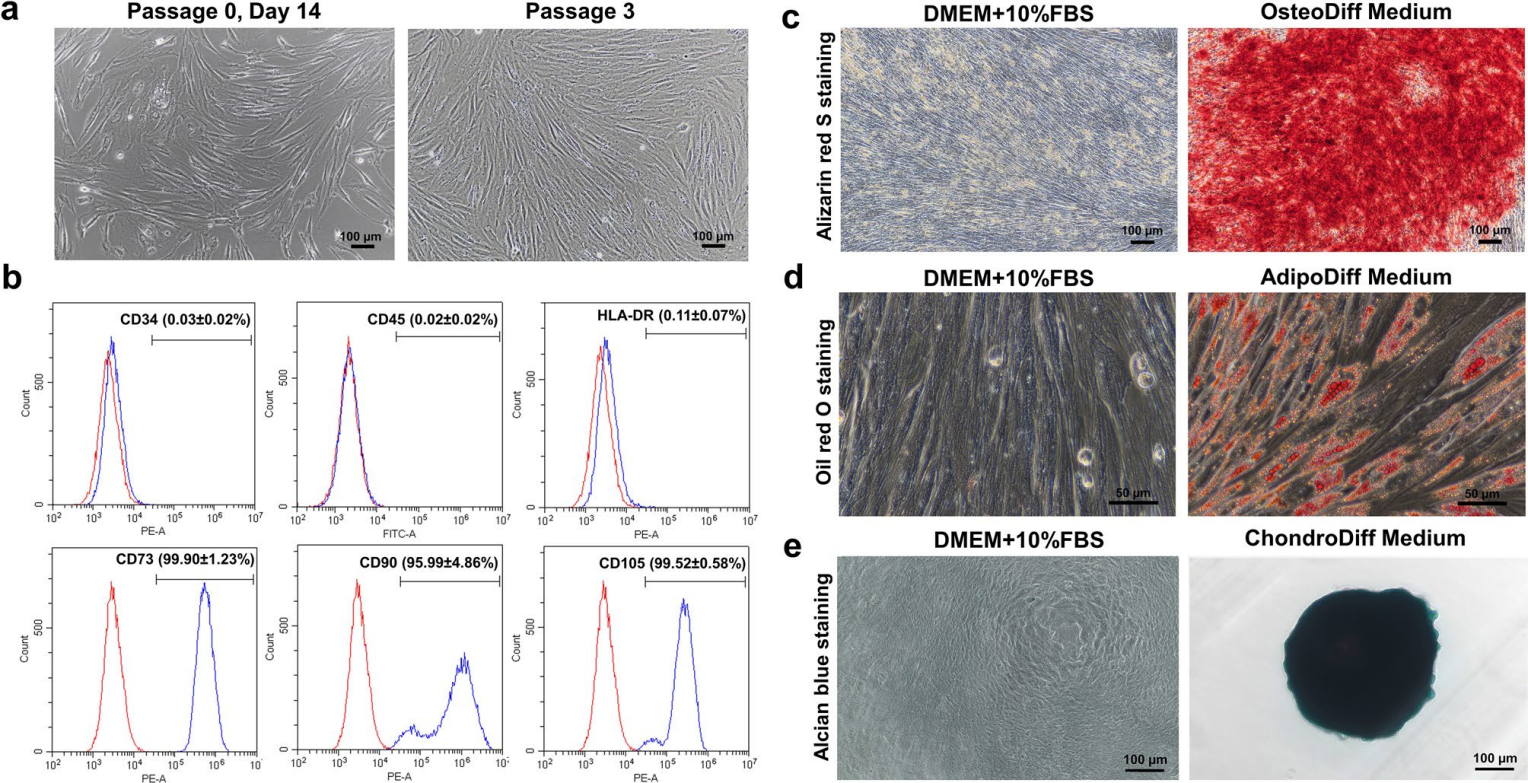
Characterization of human placenta-derived mesenchymal stem cells (PL-MSCs). (**a**) The spindle shape morphology of PL-MSCs cultured in DMEM supplemented with 10% fetal bovine serum on day 7 after removal of non-adherent cells (left) and in passages 3 (right). (**b**) Flow cytometric analysis of surface marker expression in PL-MSCs showing positive expression of MSC markers (CD73, CD90, CD105) and negative expression of hematopoietic markers (CD34, CD45, HLA-DR). (**c**) Brilliant orange-red staining of alizarin red S in PL-MSCs on day 28 of their osteogenic differentiation. (**d**) Positive signal of Oil Red O staining in PL-MSCs on day 28 on their adipogenic differentiation. (**e**) Chondrogenic differentiation potential of PL-MSCs demonstrated by Alcian positive blue color staining of positive colonies (right) that develop in the presence of chondrogenic differentiation media. Differentiated colonies were obtained from cells of all 5 donor placentas. (**a**) and (**c**) were captured with 10X magnification. Scale bar = 100 μm. (**d**) was captured with 40X magnification. Scale bar = 50 μm. (**e**) was captured with 20X magnification. Scale bar = 100 μm.

MSCs were isolated from the human placenta and cultured in DMEM supplemented with 10% FBS. After initial seeding for 7 days, non-adherent cells were removed and the isolated PL-MSCs exhibited spindle-shaped morphology. These cells were continuously cultured, maintained at 37 °C in the humidified incubator, and the medium was replaced every 3 days. Approximately 2 weeks after initial seeding, the cell density nearly reached 80% of the total area of the culture flask. From passage 3 onward, the isolated PL-MSCs rapidly proliferated and exhibited a homogeneous spindle-shaped morphology (Fig. 2a).

The expression of cell surface markers on PL-MSCs was determined using FITC-conjugated mouse anti-human CD 45 antibody and PE-conjugated mouse anti-human HLA-DR, CD34, CD73, CD90, and CD105 antibodies. At passages 3–5, PL-MSCs expressed high levels of typical MSC markers, including CD73 (99.90 ± 1.23%), CD90 (95.99 ± 4.86%), and CD105 (99.52 ± 0.58%). In contrast, they did not express surface markers associated with the hematopoietic lineage, CD34 (0.03 ± 0.02%), CD45 (0.02 ± 0.02%) or HLA-DR (0.11 ± 0.07) (Fig. 2b).

The trilineage differentiation potentials of PL-MSCs were examined by induction in osteogenic, adipogenic, and chondrogenic induction media. After 28 days in osteogenic induction medium, PL-MSCs showed signs of mineralization due to extracellular calcium deposition and were positive for alizarin red S staining. Negative control cultured in the complete DMEM medium did not have any calcium deposits and were negative for alizarin red S staining (Fig. 2c). Confirming the adipogenic differentiation potential, PL-MSCs cultured in adipogenic differentiation medium for 28 days had an increased accumulation of lipid droplets in their cytoplasm and these lipid droplets were positive for Oil Red O staining, while control cell cultured in complete DMEM medium did not have lipid droplet formation in their cytoplasm and were negative for Oil Red O staining (Fig. 2d). To confirm the chondrogenic differentiation potential of PL-MSCs, PL-MSCs were cultured in chondrogenic medium for 21 days. The typical extracellular matrix of chondrogenic differentiation was evaluated in spheroid cultures using Alcian blue staining. After incubation with a chondrogenic differentiation medium, the PL-MSCs began to form a spheroidal mass that became more prominent and condensed as time passed and was positive for Alcian blue staining on day 21. Controls cultured in the completed DMEM medium did not form a spheroidal structure and were negative for Alcian Blue staining (Fig. 2e, Supplementary Fig. 1).

The results indicated that the cells isolated from the placenta are mesenchymal stem cells that meet the criteria recommended by the International Society for Cellular Therapy. Therefore, PL-MSCs passage 3 onward were used for subsequent experiments.

### Viability of fucoxanthin-treated PL-MSCs

**Figure 3:**
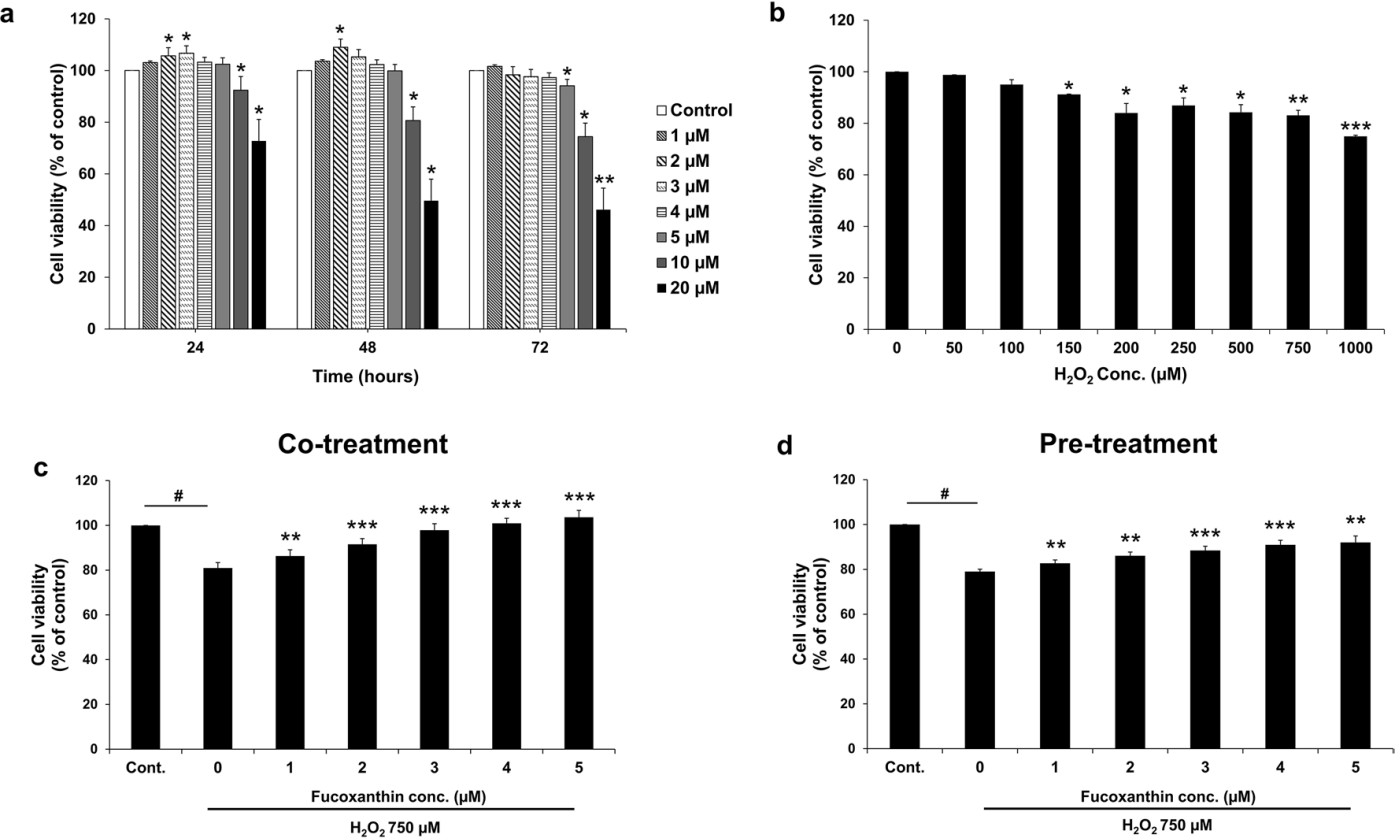
Effects of fucoxanthin on the viability of H_2_O_2_-treated PL-MSCs. (**a**) MTT assay showed the viability of PL-MSCs treated with increasing concentrations of fucoxanthin for 24–72 h. (**b**) MTT assay showed dose-dependent decreased viability of PL-MSCs treated with H_2_O_2_ for 24 h. (**c**) The viability of PL-MSCs after 24 h of co-treatment with 750 μM H_2_O_2_ and increasing concentrations (up to 5 μM) of fucoxanthin. (**d**) The viability of PL-MSCs pretreated with fucoxanthin for 24 h before H_2_O_2_ treatment for 24 h. (**a**) and (**b**) are presented as mean±SEM, n=3. The statistical significance was tested using the pair T-test. *p ≤ 0.05, **p ≤ 0.01, and ***p ≤ 0.001 vs. PL-MSCs cultured in completed DMEM medium. (**c**) and (**d**) are presented as mean ± SEM, *n* = 5. The statistical significance was tested using the pair T-test. ^#^*p* ≤ 0.001 vs. PL-MSCs cultured in completed DMEM medium. *p ≤ 0.05, **p ≤ 0.01, and ***p ≤ 0.001 vs. H_2_O_2_-treated PL-MSCs without fucoxanthin.

The MTT assay was used to determine cell viability of PL-MSCs treated with various concentrations of fucoxanthin (1, 2, 3, 4, 5, 10, and 20 µM) for 24, 48, and 72 h. Treatment of PL-MSCs with fucoxanthin at concentrations of up to 5 μM for 24 and 48 h increased their viability. However, treatment with 10 and 20 µM fucoxanthin for 24 and 48 h significantly decreased the viability of PL-MSCs. Similarly, treatment with 5, 10, and 20 µM fucoxanthin for 72 h significantly decreased the viability of PL-MSCs (Fig. 3a). Therefore, to avoid cytotoxic effects, the fucoxanthin concentration of up to 5 µM was selected for subsequent experiments.

### H_2_O_2_-induced cytotoxic effect on PL-MSCs

PL-MSCs were treated with H_2_O_2_ for 24 h at various concentrations in the range between 50 and 1000 µM to determine the optimal concentrations of H_2_O_2_ to induce oxidative damage to PL-MSCs. Cell viability was evaluated using an MTT assay. The result showed that the viability of PL-MSCs gradually decreased as the concentration of H_2_O_2_ increased. Compared to the control group, a significant decrease was observed when the concentration of H_2_O_2_ treatment was 150 µM or higher. At a maximum concentration of 1000 µM H_2_O_2_, 80% of the PL-MSCs remained viable (Fig. 3b). Consequently, a concentration of 750 µM H_2_O_2_ was selected for subsequent experiments.

### The effect of fucoxanthin on the viability of H_2_O_2_ treated PL-MSCs

Under oxidative stress conditions, PL-MSCs were treated with 1–5 µM fucoxanthin to assess the effects of fucoxanthin on the viability of PL-MSCs. Firstly, PL-MSCs were treated with 750 µM H_2_O_2_ together with 1–5 µM fucoxanthin for 24 h. The results demonstrated that the viability of PL-MSCs was significantly reduced after treatment with 750 µM H_2_O_2_ for 24 h compared to the control. In contrast, the viability of H_2_O_2_-treated PL-MSCs increased progressively with the increase in fucoxanthin concentrations. Interestingly, fucoxanthin treatment at a maximum concentration of 5 µM could restore the viability of H_2_O_2_-treated PL-MSCs to a level comparable to the control cultured in complete DMEM without H_2_O_2_ (Fig. 3c). Secondary, PL-MSCs were pre-treated with 1–5 µM fucoxanthin for 24 h before being further treated with 750 µM H_2_O_2_ for 24 h. Similarly to the co-treatment, the viability of pre-treated PL-MSCs was significantly reduced after treatment with 750 µM H_2_O_2_ for 24 h compared to the control. However, pre-treatment with fucoxanthin could significantly increase the viability of PL-MSCs in a dose-dependent manner compared to H_2_O_2_-treated PL-MSCs. Unlike the co-treatment group, pre-treatment with fucoxanthin at the maximum concentration, 5 µM, could not restore the viability of H_2_O_2_-treated PL-MSCs to a level comparable to that of the control cultured in complete DMEM without H_2_O_2_ (Fig. 3d). Overall, the findings indicated that treatment with fucoxanthin could rescue the cytotoxic effects of H_2_O_2_-induced oxidative stress on PL-MSCs.

### The effect of fucoxanthin on the replicative senescence of PL-MSCs

**Figure 4:**
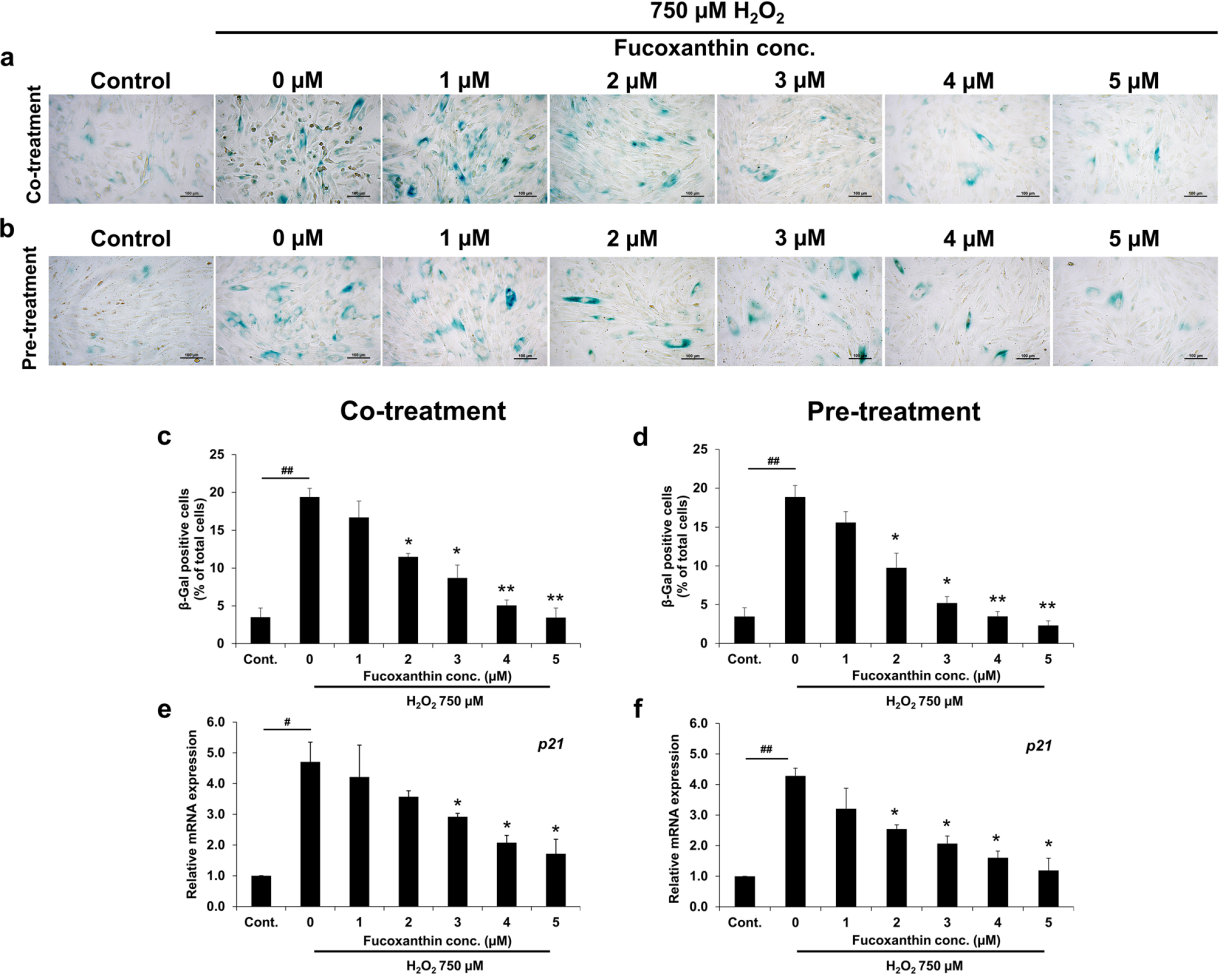
Effects of fucoxanthin on replicative senescence of H_2_O_2_-treated PL-MSCs. (**a**) The representative images of β-Gal staining of PL-MSCs after 24 h of co-treatment with 750 μM H_2_O_2_ and 1–5 μM fucoxanthin. (**b**) β-Gal staining of PL-MSCs after pre-treated with fucoxanthin for 24 h, followed by treatment with 750 μM H_2_O_2_ for 24 h. (**c**) and (**d**) Quantitation of β-Gal positive cells (% of β-Gal positive cells) of co-treated or pre-treated with 1–5 μM fucoxanthin and 750 μM H_2_O_2_ for 24 h, respectively. (**e**) Quantitative real-time RT-PCR showed the expression of *p21* in PL-MSCs after co-treatment with 750 μM H_2_O_2_ and 1–5 μM fucoxanthin for 24 h. (**f**) Quantitative real-time RT-PCR showed the expression of *p21* in PL-MSCs pre-treated with fucoxanthin for 24 h followed by treatment with H_2_O_2_ for 24 h. Data are presented as mean ± SEM, *n* = 3. Statistical significance was tested using the pair T-test. ^#^*p* ≤ 0.05, ^##^*p* ≤ 0.01 vs. PL-MSCs culture in completed DMEM medium. *p ≤ 0.05, **p ≤ 0.01 vs. H_2_O_2_-treated PL-MSCs without fucoxanthin. (**a**) and (**b**) were captured with 20X magnification. Scale bar = 100 μm.

Oxidative stress is the primary factor responsible for the replicative senescence of MSCs, which restricts their ability to proliferate. To examine the impact of fucoxanthin on the senescence of PL-MSCs, PL-MSCs were exposed to a combination of 750 µM H_2_O_2_ and 1–5 µM fucoxanthin for 24 h. The assessment of cell senescence was conducted by measuring β-Gal activity and evaluating the expression of *p21*, a senescence marker that functions as a cell cycle inhibitor. It was observed that PL-MSCs exposed to 750 µM H_2_O_2_ showed a significant increase in the proportion of cells that tested positive for β-Gal as compared to the control group (Fig. 4a–d). Administration of fucoxanthin resulted in a considerable decrease in the number of β-Gal positive cells when compared to PL-MSCs treated with H_2_O_2_ without fucoxanthin (Fig. 4a and c). In addition, the level of *p21* expression in PL-MSCs treated with H_2_O_2_ showed a significant increase of up to four-fold compared to the control group (Fig. 4e, f). Interestingly, fucoxanthin treatment reduced *p21* expression in H_2_O_2_-treated PL-MSCs in a dose-dependent manner. Furthermore, PL-MSCs treated with fucoxanthin at a concentration of 3–5 µM showed a significant reduction in *p21* expression compared to the group that did not receive fucoxanthin (Fig. 4e). Additionally, pre-treatment with fucoxanthin decreased the number of β-Gal positive cells in H_2_O_2_-treated PL-MSCs in a dose-dependent manner similar to the co-treatment condition (Fig. 4b and d). PL-MSCs that were pre-treated with fucoxanthin at concentrations of 2–5 µM showed a significant reduction in *p21* expression as compared to the group that did not receive fucoxanthin (Fig. 4f). The findings indicated that co-treatment and pre-treatment with fucoxanthin decreased the replicative senescence of H_2_O_2_-treated PL-MSCs.

### SOD activity in fucoxanthin-treated PL-MSCs

**Figure 5:**
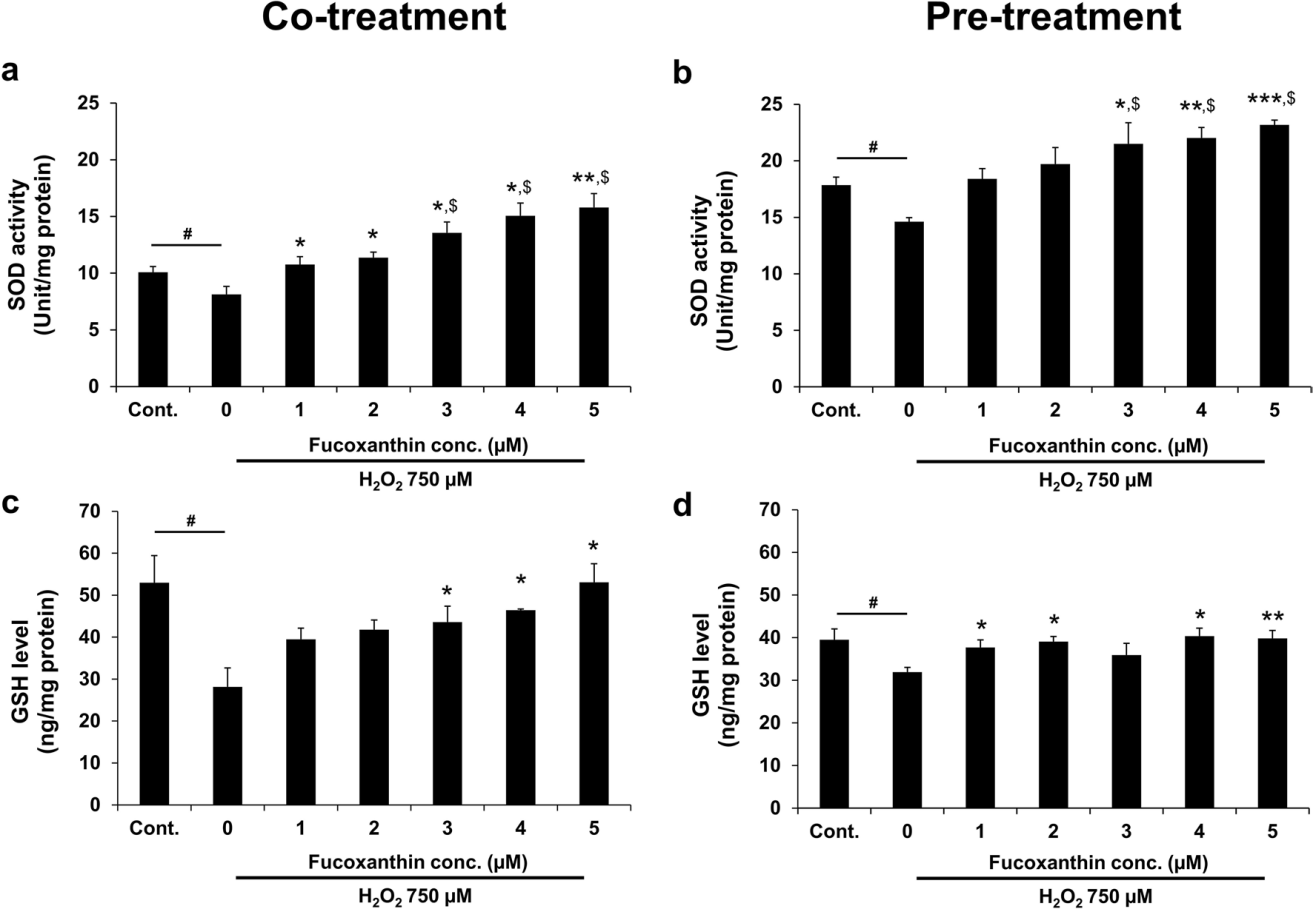
Effects of fucoxanthin on SOD activity and GSH level in H_2_O_2_-treated PL-MSCs. (**a**) SOD activity of PL-MSCs after 24 h of co-treatment with 750 μM H_2_O_2_ and fucoxanthin. (**b**) SOD activity of PL-MSCs pre-treated with fucoxanthin for 24 h followed by treatment with 750 μM H_2_O_2_ for 24 h. (**c**) GSH level of PL-MSCs after 24 h co-treatment with 750 μM H_2_O_2_ and fucoxanthin. (**d**) GSH level of PL-MSCs after fucoxanthin treatment for 24 h, followed by H_2_O_2_ treatment for 24 h. Data are presented as mean ± SEM, *n* = 4. Statistical significance was tested using the pair T-test. ^#^*p* ≤ 0.05 vs. PL-MSCs cultured in the completed DMEM medium. *p ≤ 0.05, **p ≤ 0.01, and ***p ≤ 0.001 vs. H_2_O_2_-treated PL-MSCs without fucoxanthin. ^$^*p* ≤ 0.05 vs. PL-MSCs cultured in completed DMEM medium.

The enzymes implicated in antioxidant defense against oxidative stress were investigated in fucoxanthin-treated H_2_O_2_-induced oxidative in PL-MSCs. Firstly, PL-MSCs were treated with 750 µM H_2_O_2_ together with 1–5 µM fucoxanthin for 24 h. PL-MSCs treated with H_2_O_2_ and fucoxanthin at a concentration of 3–5 µM showed a significant increase in SOD activity compared to H_2_O_2_-treated PL-MSCs without fucoxanthin and also compared to the control (without H_2_O_2_) (Fig. 5a). Similar to the co-treatment, fucoxanthin pre-treatment protocol resulted in an increased SOD activity in a dose-dependent fashion. Specifically, pre-treatment with fucoxanthin at concentrations of 3–5 µM significantly increased SOD activity compared to untreated PL-MSCs. Moreover, pre-treatment with fucoxanthin could significantly restore SOD activity in H_2_O_2_-treated PL-MSCs to a level higher than the control (Fig. 5b).

### Intracellular GSH level in fucoxanthin-treated PL-MSCs

In addition to measuring SOD activity, the intracellular GSH level was also determined in H_2_O_2_-treated PL-MSCs to assess the effect of fucoxanthin on resistance to oxidative stress in PL-MSCs treated with fucoxanthin. Treatment with 750 µM H_2_O_2_ significantly decreased intracellular GSH levels compared to the control. Fucoxanthin treatment has gradually increased GSH levels in H_2_O_2_-treated PL-MSCs in a dose-dependent manner. Specifically, fucoxanthin at a concentration of 3 and 5 µM significantly increased intracellular GSH levels compared to H_2_O_2_-treated PL-MSCs without fucoxanthin (Fig. 5c). It is interesting to note that treatment with fucoxanthin at 5 µM could restore the intracellular GSH level in H_2_O_2_-treated PL-MSCs to a level comparable to control. Similar results were observed under the pre-treatment condition; PL-MSCs pre-treated with the various concentrations of fucoxanthin had significantly higher intracellular GSH levels than untreated PL-MSCs and the treatment could restore intracellular GSH levels to the level of controls (Fig. 5d). Collectively, the results indicated that the co-treatment and pre-treatment of fucoxanthin increased SOD activities and intracellular GSH levels to protect cells from H_2_O_2_-induced oxidative damage.

### Intracellular ROS production in fucoxanthin-treated PL-MSCs

**Figure 6:**
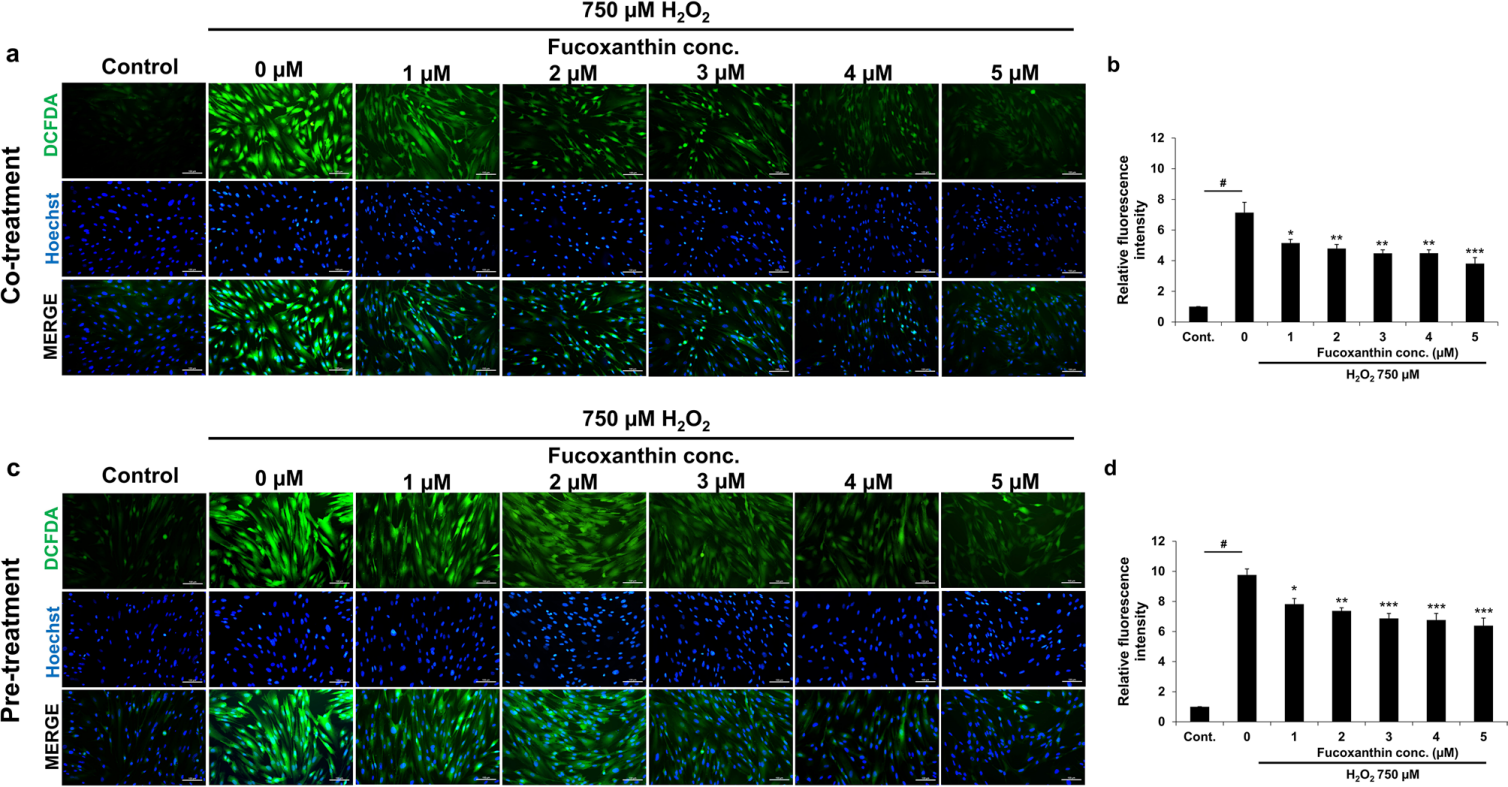
Effects of fucoxanthin on intracellular ROS production in H_2_O_2_-treated PL-MSCs. (**a**) Fluorescent micrograph illustrating the intracellular ROS content in PL-MSCs after 24 h of co-treatment with 750 μM H_2_O_2_ and fucoxanthin. (**b**) Relative fluorescence intensity of the intracellular ROS content in PL-MSCs after 24 h of co-treatment with H_2_O_2_ and fucoxanthin. (**c**) Fluorescent micrographs illustrated the intracellular ROS content in PLMSCs that were pre-treated with fucoxanthin for 24 h, followed by treatment with 750 μM H_2_O_2_ for 24 h. (**d**) Relative fluorescence intensity of the intracellular ROS content in PL-MSCs pre-treatment with fucoxanthin for 24 h, followed by treatment with H_2_O_2_ for 24 h. Data are presented as mean ± SEM, *n* = 3. Statistical significance was tested using the pair T-test. ^#^*p* ≤ 0.001 vs. PL-MSCs cultured in completed DMEM medium. *p ≤ 0.05, **p ≤ 0.01, and ***p ≤ 0.001 vs. H_2_O_2_-treated PL-MSCs without fucoxanthin. Green = DCF-DA, Blue = Hoechst33342.

To further elucidate the cytoprotective mechanisms of fucoxanthin in H_2_O_2_-induced oxidative damage in PL-MSCs, intracellular ROS production was measured. PL-MSCs were co-treated and pre-treated with 1–5 µM fucoxanthin and 750 µM H_2_O_2_ for 24 h and intracellular ROS production was evaluated using 2′,7′-dichlorofluorescein diacetate (DCFH-DA). The amounts of intracellular ROS content were measured and analyzed as fluorescence intensity relative to the control. In the co-treatment condition, the intracellular ROS content of the H_2_O_2_-treated PL-MSCs significantly increased up to 8 times compared to the control. Treatment with fucoxanthin at all concentrations significantly decreased intracellular ROS production in H_2_O_2_-treated PL-MSCs compared to untreated PL-MSCs (Fig. 6a and b). In the pre-treatment condition, intracellular ROS production in H_2_O_2_-treated PL-MSCs also decreased in a dose-dependent manner similar to the co-treatment condition (Fig. 6c and d). The results suggested that co-treatment and pre-treatment with 1–5 µM fucoxanthin decreased intracellular ROS production in H_2_O_2_-treated PL-MSCs.

### SOD expression in fucoxanthin-treated PL-MSCs

**Figure 7:**
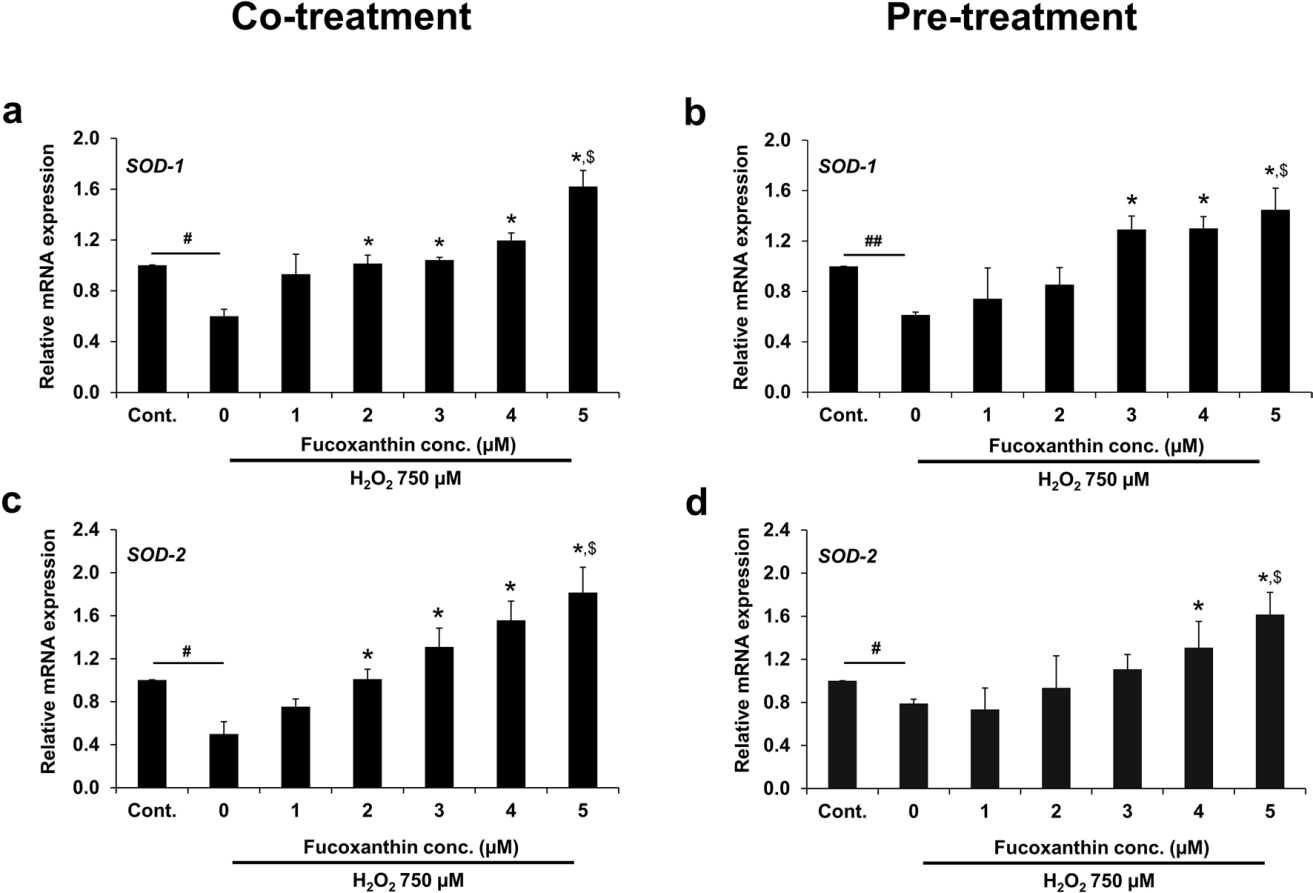
Quantitative real-time RT-PCR showed the expression of *SOD-1* and *SOD-2* in H_2_O_2_-treated PL-MSCs. (**a**) The expression of *SOD-1* in PL-MSCs after 24 h of co-treatment with 750 μM H_2_O_2_ and fucoxanthin. (**b**) The expression of *SOD-1* in PL-MSCs pre-treated with fucoxanthin for 24 h followed by treatment with 750 μM H_2_O_2_ for 24 h. (**c**) The expression of *SOD-2* in PL-MSCs after 24 h of co-treatment with 750 μM H_2_O_2_ and fucoxanthin. (**d**) The expression of *SOD-2* in PL-MSCs pre-treated with fucoxanthin for 24 h, followed by treatment with 750 μM H_2_O_2_ for 24 h. Data are presented as mean ± SEM, *n* = 3. Statistical significance was tested using the pair T-test. ^#^*p* ≤ 0.01, ^##^*p* ≤ 0.001 vs. PL-MSCs cultured in the completed DMEM medium. *p ≤ 0.05 vs. H_2_O_2_-treated PL-MSCs without fucoxanthin. ^$^*p* ≤ 0.05 vs. PL-MSCs cultured in the completed DMEM medium.

Quantitative real-time RT-PCR was used to investigate the expressions of *SOD-1* and *SOD-2*, in conjunction with the SOD activity assay. Treatment with 750 µM H_2_O_2_ for 24 h resulted in a significant reduction in the expression of *SOD-1* and *SOD-2* compared to the control while treatment with increasing concentrations of fucoxanthin resulted in a gradual up-regulation of the expressions of *SOD-1* and *SOD-2* in H_2_O_2_-treated PL-MSCs. A significant increase in *SOD-1* and *SOD-2* expressions was observed in PL-MSCs treated with fucoxanthin at 2–5 µM compared to the untreated group (Fig. 7a and c). Moreover, fucoxanthin at 2–5 µM could restore *SOD-1* and *SOD-2* expressions in H_2_O_2_-treated PL-MSCs up to a level comparable to the control. Furthermore, significantly higher *SOD-1* and *SOD-2* expressions were observed in PL-MSCs treated with fucoxanthin at 5 µM (Fig. 7a and c). Under pre-treatment conditions, PL-MSCs treated with fucoxanthin at 3–5 µM exhibited significantly higher *SOD-1* expressions than the untreated group. In contrast, fucoxanthin only at 4–5 µM significantly increased *SOD-2* expression compared to the untreated group. Furthermore, treatment with 5 µM fucoxanthin could up-regulate the expression of *SOD-1* and *SOD-2* exceeding the control (Fig. 7b and d).

### Expression of cyclin D1 in fucoxanthin-treated PL-MSCs

**Figure 8:**
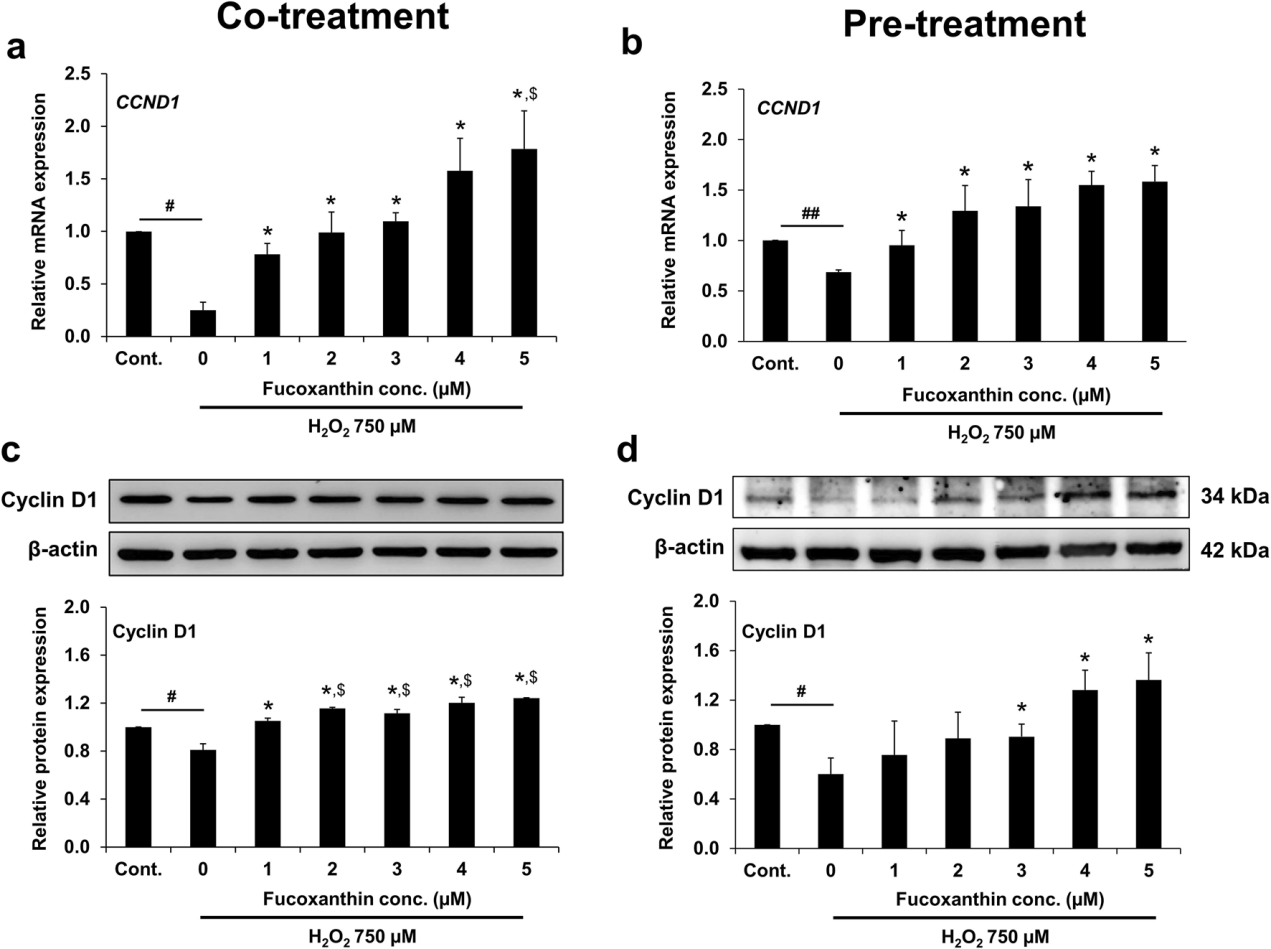
Effects of fucoxanthin on cyclin D1 expression in H_2_O_2_-treated PL-MSCs. (**a**) Quantitative real-time RT-PCR showed the expression of cyclin D1 in PL-MSCs after 24 h of co-treatment with H_2_O_2_ and fucoxanthin. (**b**) Quantitative real-time RT-PCR showed the expression of cyclin D1 in PL-MSCs pre-treated with fucoxanthin for 24 h followed by treated with H_2_O_2_ for 24 h. (**c**) Western blot analysis showed the expression of cyclin D1 in PL-MSCs after 24 h of co-treatment with H_2_O_2_ and fucoxanthin. (**d**) Western blot analysis showed the expression of cyclin D1 in PL-MSCs after pre-treatment with fucoxanthin for 24 h followed by H_2_O_2_-treatment for 24 h. The blotted membranes were cropped before hybridization with primary antibodies. The original blots of (**c**) and (**d**) are shown in Supplementary Fig. 2 and 3, respectively. Data are presented as mean ± SEM, *n* = 3. Statistical significance was tested using the pair T-test. ^#^*p* ≤ 0.05, ^##^*p* ≤ 0.01 vs. PL-MSCs culture in the completed DMEM medium. *p ≤ 0.05 vs. H_2_O_2_-treated PL-MSCs without fucoxanthin. ^$^*p* ≤ 0.05 vs. PL-MSCs cultured in the completed DMEM medium.

The expression of a cell cycle regulatory protein, cyclin D1, was examined to explore the mechanism by which fucoxanthin improves the viability of PL-MSCs under oxidative stress conditions. The expression of *cyclin D1* gene (*CCND1*) and the levels of cyclin D1 protein were examined using quantitative real-time RT-PCR and Western blotting analysis, respectively, in both PL-MSCs co-treated and pre-treated with 1–5 µM fucoxanthin and 750 µM H_2_O_2_ for 24 h. Both *CCND1* and cyclin D1 protein levels in PL-MSCs treated with 750 µM H_2_O_2_ were significantly reduced compared to the control (Fig. 8). Under co-treatment conditions, fucoxanthin-treated PL-MSCs significantly restored cyclin D1 expression, in a dose-dependent manner, compared to PL-MSCs subjected to the same oxidative stress but without fucoxanthin (Fig. 8a and c, Supplementary Fig. 2). Furthermore, treatment with fucoxanthin at 2–5 µM could significantly restore the expression of the cyclin D1 protein to a level that exceeds the control. Similarly, in fucoxanthin-pretreated PL-MSCs, cyclin D1 expression was progressively up-regulated, both at the transcriptional and protein levels, with increasing concentrations of fucoxanthin. Fucoxanthin at 1–5 µM significantly increased *CCND1* expression compared to PL-MSCs cultured in 750 µM H_2_O_2_ without fucoxanthin. However, fucoxanthin only at 3–5 µM significantly increased cyclin D1 expression compared to PL-MSCs cultured in 750 µM H_2_O_2_ without fucoxanthin. Interestingly, PL-MSCs treated with 4–5 µM fucoxanthin exhibited higher cyclin D1 expression than the control (Fig. 8b and d, Supplementary Fig. 3).

### Expression of PI3K/Akt/Nrf-2 in fucoxanthin-treated PL-MSCs

**Figure 9:**
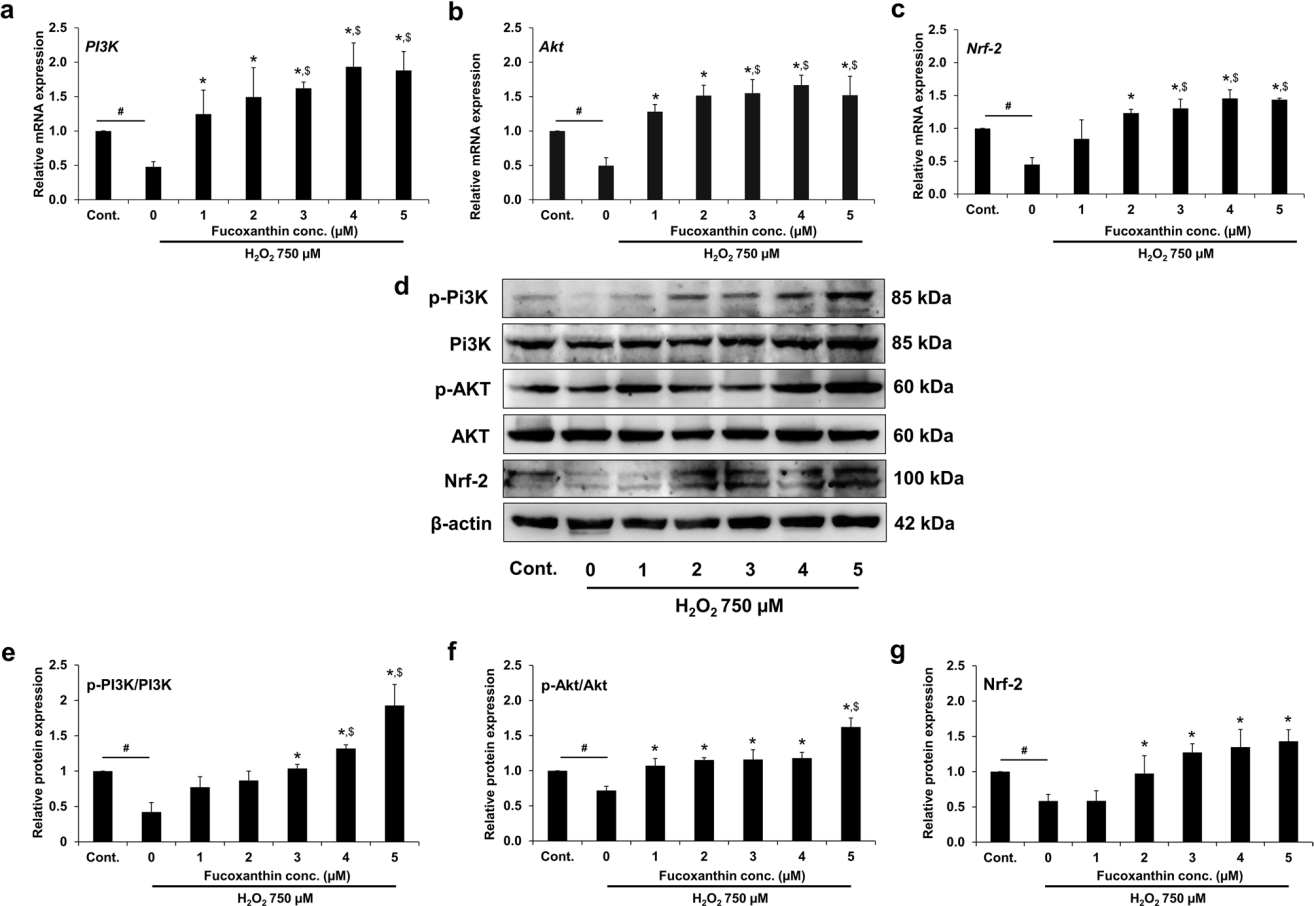
Effects of fucoxanthin on the expression of PI3K/Akt/Nrf-2 in H_2_O_2_-treated PL-MSCs. Quantitative real-time RT-PCR showed the expression of *PI3K* (**a**), *Akt* (**b**), and *Nrf-2* (**c**) in PL-MSCs after 48 h of co-treatment with 750 μM H_2_O_2_ and fucoxanthin. (**d**) Western blot analysis showed the expression of PI3K/Akt/Nrf-2. The blotted membranes were cropped before hybridization with primary antibodies. The original blots of (**d**) are shown in Supplementary Fig. 4–6. (**e**)–(**g**) Relative protein expression level of p-PI3K/PI3K, p-Akt/Akt, and Nrf-2 in PL-MSCs after 48 h of co-treatment with 750 μM H_2_O_2_ and fucoxanthin. Data are presented as mean ± SEM, *n* = 3. Statistical significance was tested using the pair T-test. ^#^*p* ≤ 0.001 vs. PL-MSCs cultured in the completed DMEM medium. *p ≤ 0.05 vs. H_2_O_2_-treated PL-MSCs without fucoxanthin. ^$^*p* ≤ 0.05 vs. PL-MSCs cultured in the completed DMEM medium.

The expression of phosphatidylinositol 3-kinase (PI3K)/Akt and the nuclear factor erythroid 2-related factor 2 (Nrf-2), as components of the cellular signaling antioxidant system, was examined in H_2_O_2_ treated PL-MSCs to gain insight into the molecular mechanism underlying the cytoprotective effects of fucoxanthin. PL-MSCs were treated with 750 µM H_2_O_2_ together with 1–5 µM fucoxanthin for 48 h. PI3K, Akt, and Nrf-2 mRNA and protein expression levels were examined using quantitative real-time RT-PCR and Western blotting analysis, respectively. In H_2_O_2_-treated PL-MSCs, the expressions of *PI3K*, *Akt,* and *Nrf-2* were significantly reduced. In contrast, treatment with increasing concentrations of fucoxanthin progressively up-regulated the expression of *PI3K*, *Akt,* and *Nrf-2* (Fig. 9a–c). PL-MSCs treated with fucoxanthin at a concentration of 2–5 µM had significantly higher *PI3K*, *Akt,* and *Nrf-2* expressions than untreated PL-MSCs. Additionally, PL-MSCs treated with fucoxanthin at a concentration of 3, 4, and 5 µM expressed higher levels of *PI3K*, *Akt,* and *Nrf-2* than control. Correspondingly, the expressions of p-PI3K/PI3K, p-Akt/Akt, and Nrf-2 proteins were gradually increased with increasing concentrations of fucoxanthin (Fig. 9d, Supplementary Fig. 4–6). Compared to H_2_O_2_-treated PL-MSCs without fucoxanthin, treatment with 2–5 µM fucoxanthin significantly increased the p-PI3K, p-Akt and Nrf-2 proteins (Fig. 9e–g). These findings suggested that the PI3K/Akt/Nrf-2 pathway is involved in the underlying mechanisms of the cytoprotective effect of fucoxanthin against H_2_O_2_-induced oxidative stress in PL-MSCs.

### Nanostring analysis of differentially expressed genes in PL-MSCs

**Figure 10:**
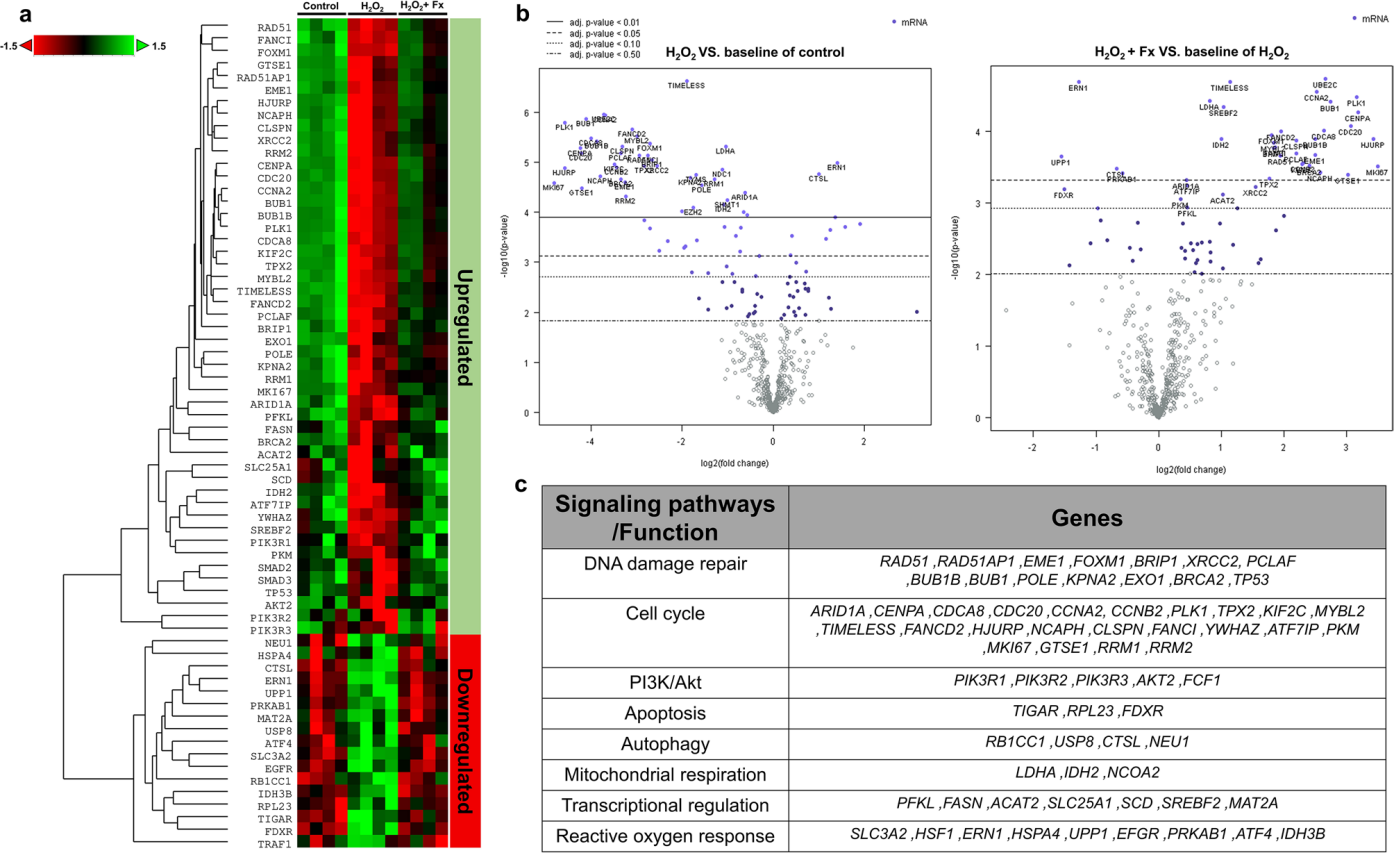
NanoString analysis of differential gene expression in PL-MSCs under oxidative stress conditions. (**a**) Heatmap of differentially expressed genes in 750 μM H_2_O_2_-treated PLMSCs and PL-MSCs treated with 750 μM H_2_O_2_ together with 5 μM fucoxanthin. (**b**) The volcano plot presents the distribution of differentially expressed genes (DE). (**c**) Summary of genes belonging to various metabolic pathways that showed statistical significance between treatment groups.

High-throughput analysis of the expression of genes involved in the cytoprotective effect of fucoxanthin was performed using NanoString nCounter through the metabolic pathway panel (Fig. 10). Fucoxanthin significantly up-regulated 43 genes in PL-MSCs treated with 750 µM H_2_O_2_ and 5 µM fucoxanthin compared to H_2_O_2_-treated PL-MSCs. The up-regulated genes were associated with 8 signaling pathways/functions including DNA damage repair, cell cycle, and proliferation, mitochondrial respiration, transcriptional regulation, reactive oxygen response, apoptosis, autophagy and the PI3K/Akt pathway (Fig. 10c). Among them, the top 20 up-regulated genes were the genes associated with cell cycle progression and DNA damage repair, for example, Antigen Ki-67 (*MKI67*), Holliday junction recognition protein (*HJURP*), cyclin A (*CCNA2*), cyclin B (*CCNB2*), cell division cycle protein 20 (*CDC20*), cell division cycle associated 8 (*CDCA*), polo-like kinase 1 (*PLK1*) and essential meiotic structure-specific endonuclease 1 (*EME1*) (Fig. 10b). It should be noted that the genes that exhibited the most substantial increases in fold change were associated with the regulation of the cell cycle. Specifically, *MKI67* (fold change = 17.82, p = 0.035), *HJURP* (fold change = 11.25, p = 0.006), and *GTSE1* (fold change = 9.88, p = 0.016) were in this category (Table 3). On the contrary, expression levels of 24 genes related to NF-κB signaling, endoplasmic reticulum (ER) stress, inflammation, autophagy, apoptosis, and the reactive oxygen response pathway were found to be down-regulated in PL-MSCs after being treated with 5 µM fucoxanthin. A notable decrease in the expression levels of endoplasmic reticulum to nucleus signaling 1 (*ERN1*), tumor necrosis factor receptor-associated factor 1 (*TRAF1*), TP53-inducible glycolysis and apoptosis regulator (*TIGAR*) and cathepsin L (*CTSL*) was observed. The analysis revealed a notable reduction in the fold change in genes related to reactive oxygen response and apoptosis, including UPP1 (fold change = -3.030, p = 0.003), FDXR (fold change = − 2.890, p = 0.003), and ERN1 (fold change = 2.50, p = 0.001) (Table 3). These evidences were consistent with a cytoprotective effect of fucoxanthin, suggesting that the compound increases PL-MSC viability by up-regulating the expression of genes involved in the cell cycle, DNA damage repair, and the PI3K/Akt pathway. Moreover, fucoxanthin treatment down-regulated the expression of genes involved in autophagy, apoptosis, oxidative stress pathways, and inflammation.

**Table 3:** Fold change in gene expression of fucoxanthin-treated PL-MSCs compared to untreated PL-MSCs under oxidative stress.

Up-regulated Genes	Gene description	Fold change	P value
*MKI67*	*Antigen Ki-67*	17.82	0.035
*HJURP*	*Holliday junction recognition protein*	11.25	0.006
*GTSE1*	*G2 And S-Phase Expressed 1*	9.88	0.016
*PLK1*	*Polo-like kinase 1*	8.60	0.001
*CENPA*	*Centromere Protein A*	8.10	0.003
*CDC20*	*Cell division cycle protein 20*	7.85	0.003
*NCAPH*	*Non-SMC Condensin I Complex Subunit H*	6.71	0.013
*BUB1*	*BUB1 mitotic checkpoint serine/threonine kinase*	6.16	0.001
*UBE2C*	*Ubiquitin conjugating enzyme E2 C*	5.73	0.002
*EME1*	*Essential meiotic structure-specific endonuclease 1*	5.68	0.015

Down-regulated Genes	Gene description	Fold change	P value
*UPP1*	*Uridine phosphorylase 1*	− 3.030	0.003
*FDXR*	*Ferredoxin Reductase*	− 2.890	0.003
*PNP*	*Purine Nucleoside Phosphorylase*	− 2.700	0.044
*ERN1*	*Endoplasmic reticulum to nucleus signaling 1*	− 2.500	0.001
*RPS6KA1*	*Ribosomal protein S6 kinase alpha 1*	− 2.140	0.028
*SLC3A2*	*Solute carrier family 3 member 2*	− 2.040	0.008
*SQSTM1*	*Sequestosome 1*	− 1.930	0.007
*TRAF1*	*Tumor necrosis factor receptor-associated factor 1*	− 1.830	0.047
*TIGAR*	*TP53-inducible glycolysis and apoptosis regulator*	− 1.770	0.017
*CTSL*	*Cathepsin L*	− 1.650	0.010

## Discussion

The results of this study indicate that the administration of fucoxanthin to PL-MSCs has a restorative effect on cell viability that was previously compromised by H_2_O_2_ exposure. Fucoxanthin was observed to slow the generation of intracellular ROS, thus protecting cells against H_2_O_2_-induced cellular damage. Furthermore, it was found to increase the activities of antioxidant enzymes, SOD, and GSH through the PI3K/Akt/Nrf-2 signaling pathways.

Mesenchymal stem cells possess multipotent properties that enable them to facilitate the restoration of tissue function after injury^32^. Due to their capacity for self-renewal and multilineage differentiation, MSC-based therapy has attracted much interest in regenerative medicine^33,34^. MSCs have been widely studied in both animal models and clinical trials^35^. With benefits in immunologic privilege, fewer ethical problems and the ability to differentiate effectively along an osteogenic lineage, they present a possible choice for use in bone regeneration and repair. Previously, the use of MSCs for fracture repair has been successfully investigated using animal models^36^. For therapeutic applications, the cell source for MSC-based therapy must be abundant and easily accessible^37^. Bone marrow-derived MSCs (BM-MSCs) are one of the MSC sources that have undergone extensive research^38^. However, there are still limitations that restrict their application in the clinical setting. These issues include the small number of cells, the fact that increasing donor age decreases proliferative and differentiation capacity, and the invasive process for cell harvesting^3,39,40^. In contrast, placenta-derived MSCs (PL-MSCs) are an attractive cell source due to their quantity and accessibility without invasive procedures^41,42^. When PL-MSCs are compared to BM-MSCs, it is observed that the former exhibit a more youthful phenotype. Additionally, PL-MSCs exhibit superior long-term growth capacity compared to BM-MSCs, along with a commensurate enhancement in plasticity^43^. The decline in activities and functions of tissue-derived MSCs with aging poses significant challenges for clinical applications in regenerative medicine^44^. Furthermore, it has been observed that PL-MSCs exhibit prolonged morphological stability during in vitro passages and retain their immune-privileged status even after differentiation into multiple lineages^31,45^. Furthermore, PL-MSCs have been the subject of numerous clinical trials and researchs^46–48^. Therefore, this study used PL-MSCs as a paradigm for a compelling source of MSC translation into regenerative medicine. The isolated PL-MSCs have been found to meet all the criteria for MSCs established by the International Society of Cellular Therapy^1^. These criteria include adherence to culture flasks, a spindle-shaped morphology, and high expression levels of MSC markers such as CD73, CD90, and CD105. On the contrary, the markers of hematopoiesis, namely CD34, CD45, and HLA-DR, were absent. Furthermore, they have the capability to undergo differentiation into osteoblasts, adipocytes, and chondrocytes.

Although MSCs have been used with some success in the clinic, there is room for improvement to reach their full clinical potential. Firstly, MSCs are rare cells in vivo and must be expanded ex vivo to generate a sufficient number of cells for clinical applications. However, MSCs undergo replicative senescence, which limits their proliferative capacity. Furthermore, this replicative senescence also compromises their immunomodulatory and differentiation functions and possibly their clinical activity against GvHD and other inflammatory pathologies^49,50^. A previous study showed that the viability of transplanted MSCs in areas of injury is limited as a result of apoptosis, which is induced by oxidative stress and hypoxic microenvironmental circumstances. This phenomenon leads to a disparity between the mechanisms responsible for antioxidant defense and the production of reactive oxygen species^51–53^. To improve the efficacy of cell therapy, it is therefore necessary to modulate MSCs in a way that improves their survival against environmental insults such as oxidative stress. It is of utmost importance to prevent oxidative stress-induced injury of transplanted MSCs and to improve their survival rate in post-transplantation. Hence, it is imperative to devise a methodology to manipulate MSCs to mitigate ROS levels both during the cell culture expansion phase and in the damaged tissue microenvironment. This approach will facilitate MSC engraftment and increase the protection of transplanted MSCs before and during transplantation, thus amplifying the efficacy of MSC-based therapy in the field of regenerative medicine.

Fucoxanthin, a xanthophyll carotenoid abundant in brown seaweed, has a strong antioxidant capacity due to a unique chemical structure that confers its biological effects. It has a polyene chain with nine conjugated double bonds and an unusual allenic bond and 5,6-monoepoxide in its molecule^13,54^. These features make fucoxanthin more effective at quenching singlet oxygen and scavenging free radicals than other carotenoids. Fucoxanthin has been shown to have the ability to reinstate cell viability in the presence of oxidative stress by reducing intracellular ROS and increasing the expression and activity of antioxidant enzymes. This phenomenon has been observed in both normal human liver cell lines^19^ and human retinal pigment epithelial cells^55^. However, the potential advantageous impacts of fucoxanthin on human MSCs have not yet been documented. The present study examined the impact of co-treatment and pre-treatment with varying concentrations of fucoxanthin on PL-MSCs that were subjected to H_2_O_2_ treatment. The results evidenced that fucoxanthin exhibited a dose-dependent effect in promoting the viability and proliferation of PL-MSCs under oxidative stress conditions, where cells were negatively affected by H_2_O_2_.

Hydrogen peroxide is a common source of oxidative stress that damages cells and tissues through ROS production and the exhaustion of antioxidants^56^. Several studies have shown that H_2_O_2_-induced oxidative stress affects various aspects of MSC properties, including apoptosis, senescence, mitochondrial dysfunction, DNA damage and proteomic alterations. These effects have the potential to reduce the viability, proliferation, migration, and differentiation potential of MSCs^57,58^, while their therapeutic effectiveness may be limited due to vulnerability to oxidative stress.

Oxidative stress is known to play a role in stem cell self-renewal and differentiation^58^. It affects both ex vivo culture expansion and longevity of MSCs, which has implications for cell therapy. Mesenchymal stem cells experience replicative senescence and decreased proliferation as they are continuously cultured and expanded ex vivo^59^. The process of aging and senescence is associated with an increased level of oxidative stress, which imposes restrictions on the number of passages and the quality of MSCs^60^.

Reactive oxygen species are generated primarily from mitochondria. High ROS levels cause cellular damage and dysfunction. Antioxidant enzymes such as SOD-1, SOD-2, and GSH exhibit potent antioxidant properties that protect cells against oxidative stress^61^. The expression of these enzymes is observed to decrease in cells that are exposed to an excessive amount of oxidative stress, leading to the accumulation of ROS within the cell^62^. The findings of the present study indicate that the administration of fucoxanthin leads to a reduction in the accumulation of intracellular ROS and a rise in the expression of antioxidant genes, namely SOD-1, SOD-2, and GSH, in PL-MSCs under both pre-treatment and co-treatment conditions.

Fucoxanthin can increase the level of GSH mRNA and protein expression through the Akt/Nrf-2/GSH-dependent antioxidant response^16^. The PI3K/Akt pathways regulate the cytoprotective effect of fucoxanthin on PL-MSCs, as indicated by the observed alteration in the phosphorylation of PI3K and Akt. Notably, the level of p-Akt expression exhibited a decrease after H_2_O_2_ exposure, which was found to be reversible after fucoxanthin treatment. PI3K/Akt signaling pathways have been widely acknowledged to have crucial regulatory functions in the regulation of various cellular behaviors of MSCs, including, but not limited to, survival, proliferation, and differentiation^20,21^.

Nrf-2 is a transcription factor that plays a crucial role in protection against oxidative stress by regulating the transcriptional activity of more than 2000 genes, mainly involved in cytoprotection^63^. Furthermore, it has been observed that Nrf-2 induces cell cycle arrest by downregulating Cyclin D1 expression^64^. Previous studies have demonstrated that the Akt/Nrf-2 pathway is integral to the fundamental mechanism underlying the antioxidant properties of fucoxanthin^15,16,19,65^. The current results showed that the administration of fucoxanthin during oxidative stress conditions resulted in up-regulation of Nrf-2, which functions as a downstream signaling molecule of PI3K/Akt. Therefore, the findings further confirm that fucoxanthin administration leads to an improvement in the survival rate of PL-MSCs, which is facilitated by activation of the PI3K/Akt/Nrf-2 signaling pathways.

Oxidative stress instigates alterations in the expression of various target genes in MSCs^66^. The phenomenon of oxidative stress has the potential to damage DNA, leading to genetic instability in cells, ultimately resulting in cell cycle arrest and apoptosis^67^. Numerous studies have demonstrated that antioxidants possess the ability to protect against the detrimental impact of oxidative stress^68,69^. The present study employed a NanoString nCounter analysis to identify genes involved in metabolic pathways and showed that the administration of fucoxanthin affects the molecular profiling of PL-MSCs that were subjected to H_2_O_2_ treatment. Specifically, the intervention resulted in the up-regulation of numerous genes that are linked to cellular survival, such as those involved in DNA damage repair and cell cycle progression. A previous study demonstrated similar results in gene expression related to pathways associated with cell cycle progression, including *CCNA*, *CCNB*, *CCND*, *PLK1* and *CDC20*^68^. In line with this, the present study demonstrated that fucoxanthin exhibited an up-regulation effect on TP53, a transcription factor that governs the genomic stability, proliferation, and differentiation of MSCs^18^. On the other hand, fucoxanthin exhibited a down-regulatory effect on genes associated with autophagy, apoptosis, oxidative stress pathways, and inflammation. Taken together, the findings indicate that the cytoprotective compound fucoxanthin has the potential to mitigate cellular damage and inhibit apoptosis in the presence of oxidative stress.

## Conclusion

This study has revealed the cytoprotective properties of fucoxanthin on PL-MSCs when subjected to oxidative stress conditions. The viability of PL-MSCs increased after treatment with fucoxanthin, which was attributed to the increased expression and activity of the antioxidant enzymes, SOD and GSH. Fucoxanthin also led to a reduction in intracellular ROS production during H_2_O_2_-induced oxidative stress through the signal cascades of PI3K/Akt/Nrf-2. These findings suggest that fucoxanthin is a potential cytoprotective agent that could be used in conjunction with MSC-based therapy for applications in regenerative medicine.

### Supplementary Information

Supplementary Figures.

## Data Availability

The data generated or analyzed during this study are included in this article, or if absent are available from the corresponding author upon reasonable request.

## Abbreviations

Akt: Protein kinase B
CAT: Catalase
CCND1: Cyclin D1
CD: Cluster of differentiation
cDNA: Complementary deoxyribonucleic acid
CO_2_: Carbon dioxide
DCFH-DA: 2′,7′-Dichlorofluorescein diacetate
DMEM: Dulbecco’s modified Eagle’s medium
DMSO: Dimethyl sulfoxide
MTT: 3-(4,5-Dimethylthiazol-2-yl)-2,5- diphenyl tetrazolium bromide
FBS: Fetal bovine serum
FITC: Fluorescein isothiocyanate
GAPDH: Glyceraldehyde-3-phosphate dehydrogenase
GSH: Glutathione
H_2_O_2_: Hydrogen peroxide
BM-MSCs: Bone marrow-derived mesenchymal stem cells
MSCs: Mesenchymal stem cells
Nrf-2: Nuclear factor erythroid 2-related factor 2
PBS: Phosphate buffered saline
PE: Phycoerythrin
PI3K: Phosphoinositide 3-kinase
PL-MSCs: Placenta-derived mesenchymal stem cells
ROS: Reactive oxygen species
SEM: Standard error of means
SOD: Superoxide dismutase
qRT-PCR: Quantitative reverse transcription polymerase chain reaction
