# Supplementary material for: Fucoxanthin diminishes oxidative stress damage in human placenta-derived mesenchymal stem cells through the PI3K/Akt/Nrf-2 pathway
Source: Sci Rep. 2023 Dec 27;13:22974. doi: 10.1038/s41598-023-49751-5 (PMC10752906; doi:10.1038/s41598-023-49751-5)
Supplement: Supplementary file 1 — Supplementary Figures. [file 41598_2023_49751_MOESM1_ESM.docx]

**Supplementary figures and figure legends**


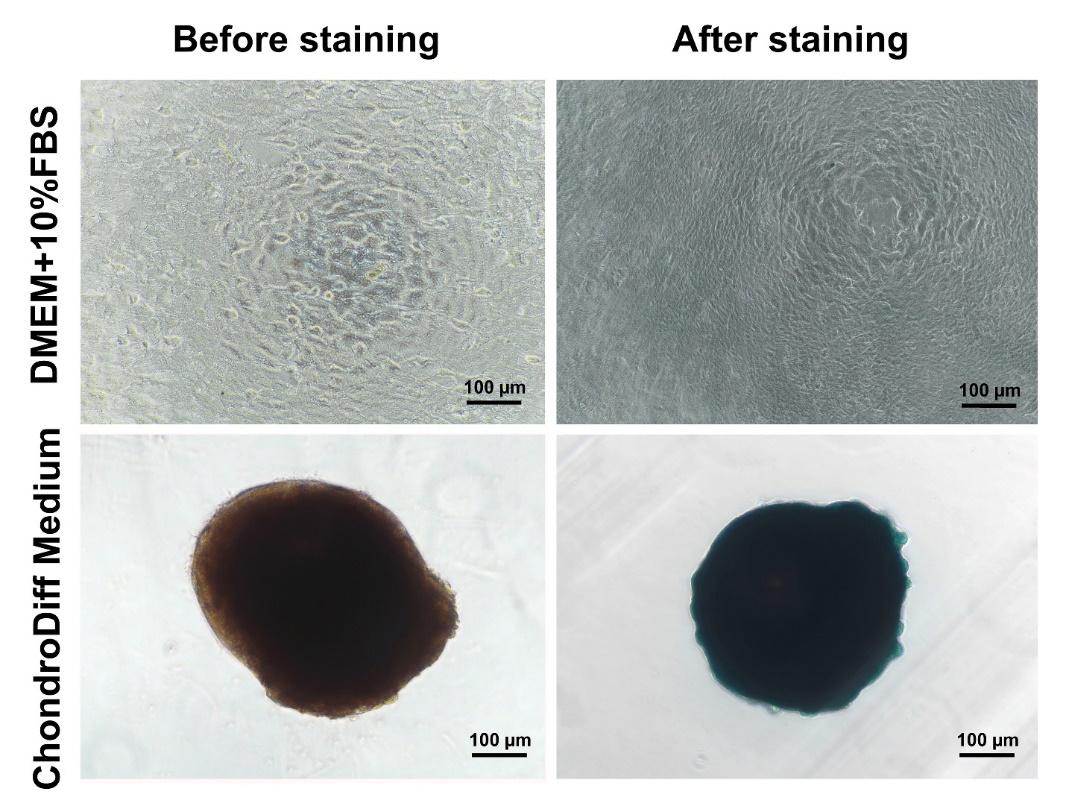


**Supplementary Figure 1** Chondrogenic differentiation potential of PL-MSCs cultured in chondrogenic differentiation medium compared to those cultured in complete medium. Differentiated cells exhibited a blue color after staining with Alcian blue. Scale bar = 100 µm.


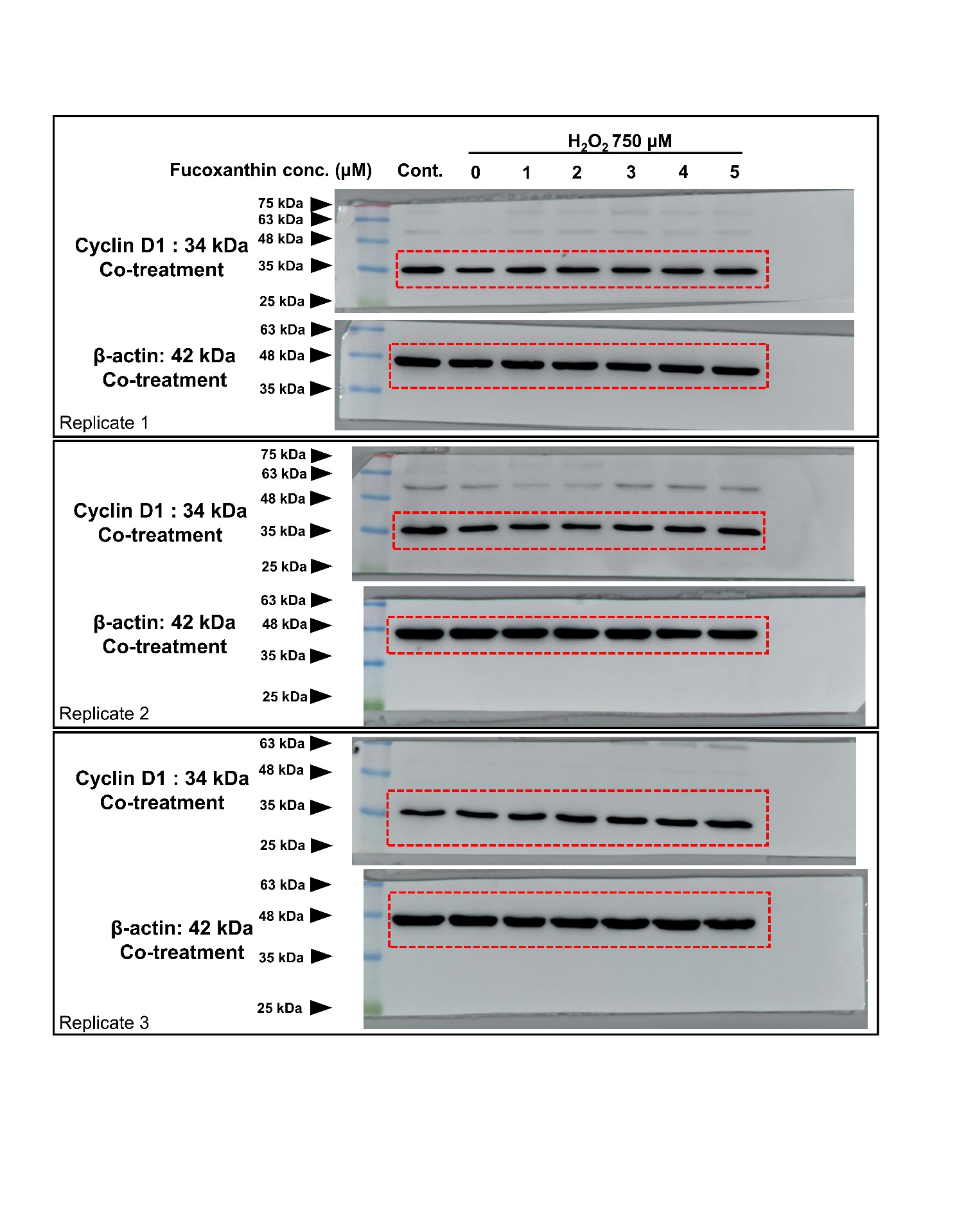


**Supplementary Figure 2** Original blots showed the expression of cyclin D1 in PL-MSCs after co-treatment with 750 µM H_2_O_2_ and various concentrations of fucoxanthin for 24 h. The blotted membranes were cut before hybridization with primary antibodies. Three replicate blots were performed. Human β-actin (MW=42 kDa) was used for normalization of protein loading.


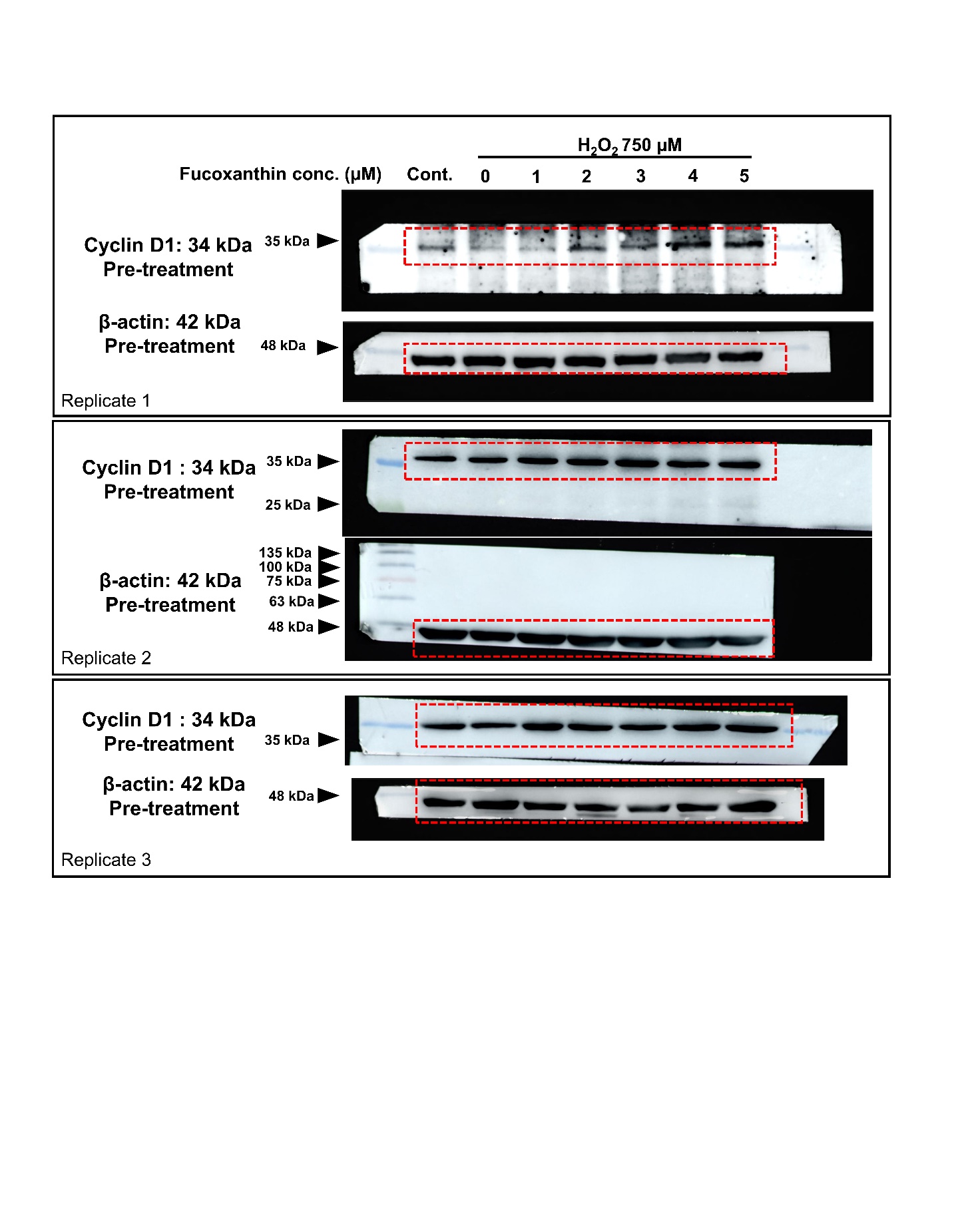


**Supplementary Figure 3** Original blots showed the expression of cyclin D1 in PL-MSCs after pre-treatment with various concentrations of fucoxanthin followed by treatment with H_2_O_2_ for 24 h. The blotted membranes were cut before hybridization with primary antibodies. Three replicate blots were performed. Human β-actin (MW=42 kDa) was used for the normalization of protein loading.


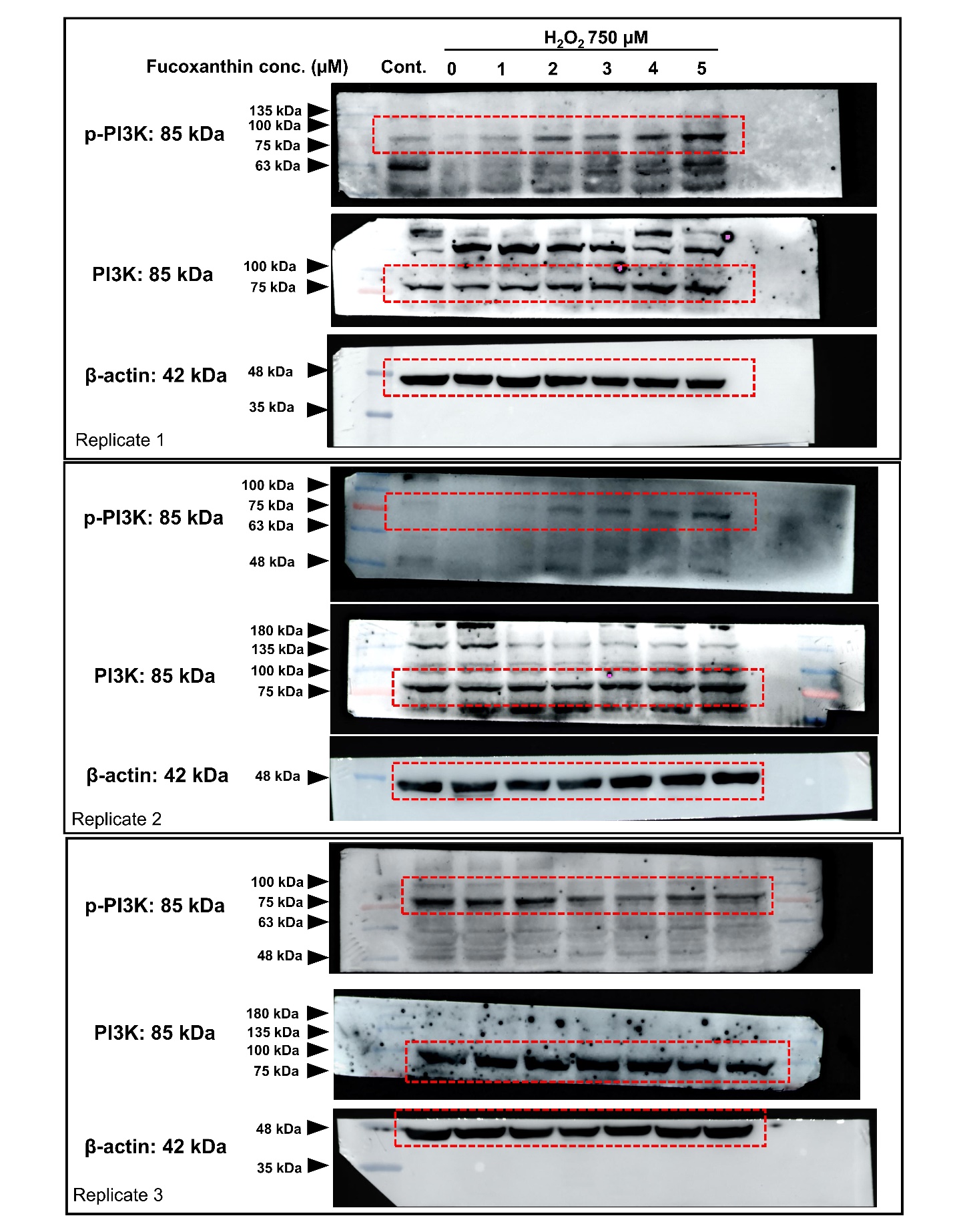


**Supplementary Figure 4** Original blots showed the expression of p-PI3K/PI3K in PL-MSCs after co-treatment with 750 µM H_2_O_2_ and various concentrations of fucoxanthin for 48 h. The blotted membranes were cut before hybridization with primary antibodies. Three replicate blots were performed. Human β-actin (MW=42 kDa) was used for normalization of protein loading.


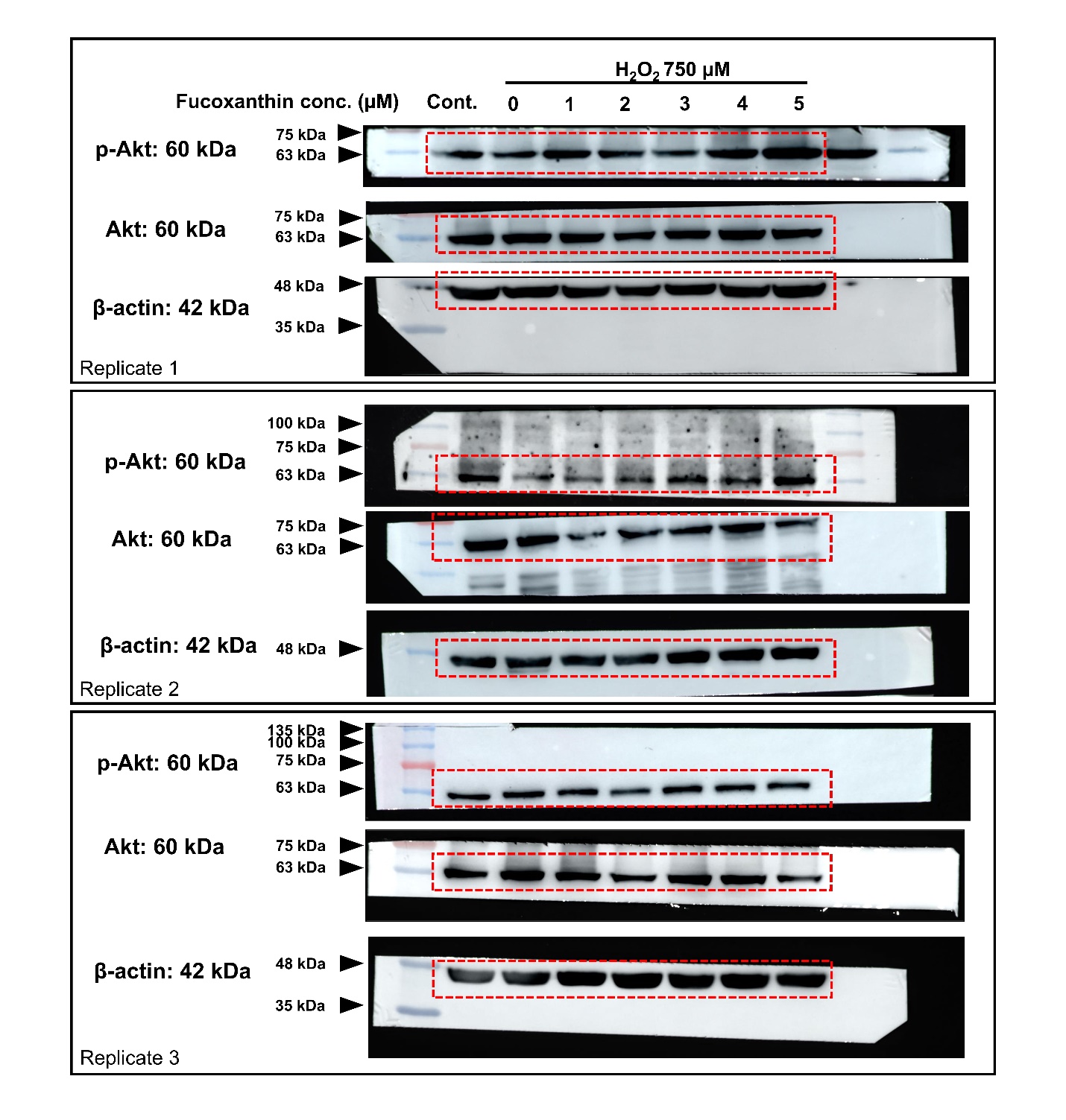


**Supplementary Figure 5** Original blots showed the expression of p-Akt/Akt in PL-MSCs after co-treatment with 750 µM H_2_O_2_ and various concentrations of fucoxanthin for 48 h. The blotted membranes were cut before hybridization with primary antibodies. Three replicate blots were performed. Human β-actin (MW=42 kDa) was used for normalization of protein loading.


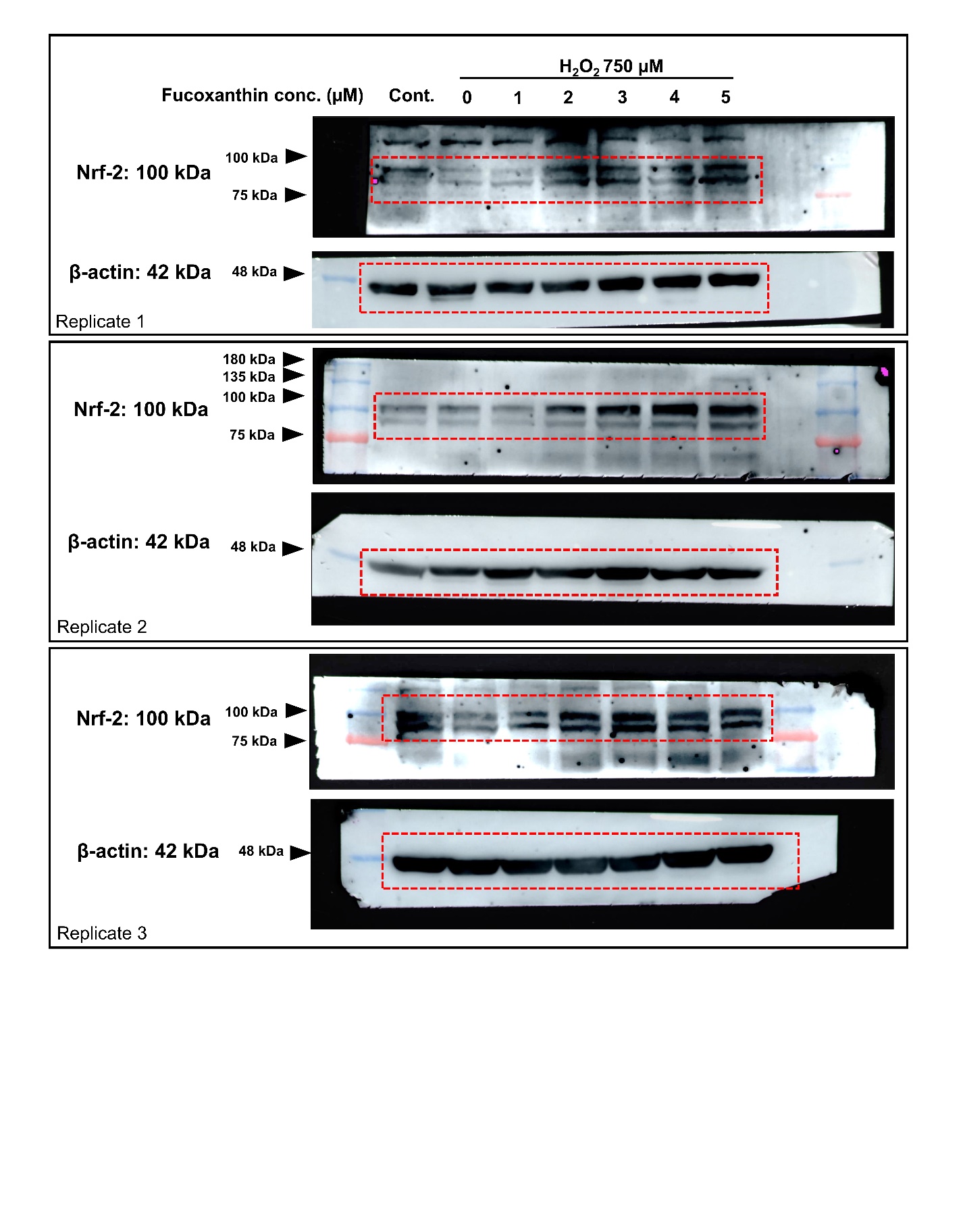


**Supplementary Figure 6** Original blots showed the expression of Nrf-2 in PL-MSCs after co-treatment with 750 µM H_2_O_2_ and various concentrations of fucoxanthin for 48 h. The blotted membranes were cut before hybridization with primary antibodies. Three replicate blots were performed. Human β-actin (MW=42 kDa) was used for normalization of protein loading.
